# Identification and Validation of a Novel Signature Based on NK Cell Marker Genes to Predict Prognosis and Immunotherapy Response in Lung Adenocarcinoma by Integrated Analysis of Single-Cell and Bulk RNA-Sequencing

**DOI:** 10.3389/fimmu.2022.850745

**Published:** 2022-06-10

**Authors:** Peng Song, Wenbin Li, Lei Guo, Jianming Ying, Shugeng Gao, Jie He

**Affiliations:** ^1^Department of Thoracic Surgery, National Cancer Center/National Clinical Research Center for Cancer/Cancer Hospital, Chinese Academy of Medical Sciences and Peking Union Medical College, Beijing, China; ^2^Department of Pathology, National Cancer Center/National Clinical Research Center for Cancer/Cancer Hospital, Chinese Academy of Medical Sciences and Peking Union Medical College, Beijing, China

**Keywords:** single-cell RNA-sequencing, NK cell marker genes, prognostic signature, immunotherapy, lung adenocarcinoma

## Abstract

Natural killer (NK) cells, the effectors of the innate immune system, have a remarkable influence on cancer prognosis and immunotherapy. In this study, a total of 1,816 samples from nine independent cohorts in public datasets were enrolled. We first conducted a comprehensive analysis of single-cell RNA-sequencing data of lung adenocarcinoma (LUAD) from the Gene Expression Omnibus (GEO) database and determined 189 NK cell marker genes. Subsequently, we developed a seven-gene prognostic signature based on NK cell marker genes in the TCGA LUAD cohort, which stratified patients into high-risk and low-risk groups. The predictive power of the signature was well verified in different clinical subgroups and GEO cohorts. With a multivariate analysis, the signature was identified as an independent prognostic factor. Low-risk patients had higher immune cell infiltration states, especially CD8^+^ T cells and follicular helper T cells. There existed a negative association between inflammatory activities and risk score, and the richness and diversity of the T-cell receptor (TCR) repertoire was higher in the low-risk groups. Importantly, analysis of an independent immunotherapy cohort (IMvigor210) revealed that low-risk patients had better immunotherapy responses and prognosis than high-risk patients. Collectively, our study developed a novel signature based on NK cell marker genes, which had a potent capability to predict the prognosis and immunotherapy response of LUAD patients.

## Introduction

Lung cancer is the global leading cause of cancer-related mortality (1), among which lung adenocarcinoma (LUAD) represents the main histological subtype, comprising nearly 50% of all lung cancers (2–4). Despite the significant advances in therapeutic strategies for LUAD, the 5-year overall survival (OS) of LUAD remains below 20% (5). Recently, the clinical application of immunotherapies targeting immune checkpoints has dramatically improved clinical benefits and shifted the treatment paradigm of LUAD (6, 7). Several biomarkers are now widely used in clinical practice to predict immunotherapy response, including PD-L1 expression and tumor mutation burden (TMB) (8). However, these biomarkers could not fully reflect the heterogeneous tumor microenvironment (TME) and clinical benefits from immunotherapy is still limited to a portion of LUAD patients (9). As a result, it is imperative to develop prediction models and identify new biomarkers to predict prognosis and therapeutic effect.

Tumor cells are surrounded by the TME, which is quite complex and comprises different immune cells, stromal cells, extracellular matrix molecules, and various cytokines (10, 11). Emerging evidence has demonstrated that the components of the TME are recognized to play vital roles in tumor initiation and progression. Furthermore, abnormal changes in TME not only impact the prognosis of patients but could also be used as a biomarker for immunotherapy (12). In the context of anti-tumor immunity, the focus is mainly on the adaptive T-cell response, while the role of innate immune cells has not yet received enough attention. Natural killer (NK) cells, a subtype of innate immune cells, can rapidly recognize and kill tumor cells (13). The efficient activity of NK cells depends entirely on a balance of inhibitory and activating receptors that can interact with ligands on target cells (14). NK cells can participate in anti-tumor immunity in the early presence of tumors by directly killing tumor cells and promoting adaptive T-cell immunological responses (15), thereby limiting tumor cell aggressiveness (16). NK and T cells work together to control cancer progression, indicating the importance of NK cells in shaping anti-tumor immunity, which has also been demonstrated by several previous studies. Reduced NK cell activity in the peripheral blood increases the risk of malignancy (17). Additionally, the higher abundance of tumor-infiltrating NK cells was significantly linked with better prognosis in different types of tumors (18–21). Given the roles of NK cells in immunity, previous studies have investigated the molecular characteristics of NK cells in infectious diseases and cancers (22–27), whereas a comprehensive molecular analysis of NK cells in LUAD is relatively poorly known.

The development of single-cell RNA-sequencing (scRNA-seq) technology and associated methods for data analysis has provided an unprecedented opportunity to unravel the molecular characteristics of diverse immune cell populations in the TME (28). Previous studies have reported that exploring gene expression signatures based on molecular characteristics of immune cells derived from scRNA-seq data might be a potent method to predict the prognosis and immunotherapy response of cancer patients (29, 30). In this study, we first performed a comprehensive analysis of scRNA-seq of LUAD to dissect the molecular characteristics of tumor-infiltrating NK cells and identify the marker genes of NK cells. Next, a NK cell marker gene signature (NKCMGS) was constructed for prognosis prediction of LUAD through bulk RNA-seq analysis. Furthermore, the predictive power of the NKCMGS was validated in six independent cohorts from the Gene Expression Omnibus (GEO) database, and the relationship between the NKCMGS and immunotherapy response in LUAD was investigated.

## Materials and Methods

### Data Collection

Totally, 1,816 samples were enrolled in this study, namely, 11 LUAD samples with scRNA-seq data, 500 LUAD samples from the TCGA, 1,007 LUAD samples from six independent GEO cohorts (https://www.ncbi.nlm.nih.gov/geo/), and 298 samples treated with immunotherapy from the IMvigor210 cohort. Single-cell RNA-sequencing data from 11 primary LUAD samples of GSE131907 were obtained from the GEO database, and were used to determine the NK cell marker genes of LUAD. The Cancer Genome Atlas (TCGA) bulk tumor transcriptomic data (FPKM normalized) and clinical information of 500 patients with LUAD were downloaded from the UCSC Xena (https://xenabrowser.net/) for identifying survival-related genes and constructing prognostic signatures. Six independent microarray datasets, namely, GSE30219 (n = 83), GSE3141 fimmu.2022.850745(n = 58), GSE50081 (n = 127), GSE26939 (n = 115), GSE72094 (n = 398), and GSE31210 (n = 226), were also obtained from the GEO database for external validation. In this study, the TCGA RNA-sequencing data of were converted into transcripts per kilobase million (TPM) values, which are more comparable between TCGA samples and microarrays (31). Transcriptomic and matched clinical data of patients who received anti-PD-L1 treatment from the IMvigor210 cohort were collected from http://research-pub.gene.com/IMvigor210CoreBiologies to explore the value of NKCMGS in speculating on the immunotherapy response (32). The study used publicly available datasets with preexisting ethics approval from original studies.

### Identification of NK Cell Marker Genes by scRNA-seq Analysis

We conducted an analysis of scRNA-seq data by R packages, including “Seurat” and “SingleR” (33). To retain high-quality scRNA-seq data, three filtering measures were applied to the raw matrix for each cell: only genes that were expressed in at least 5 single cells were included, cells that expressed less than 100 genes were eliminated, and cells with more than 5% of mitochondrial genes were also removed. We first used the “Seurat” R package to normalize scRNA-seq data by the “NormalizeData” function, setting the normalization method as “LogNormalize.” Normalized scRNA-seq data were then transformed into a Seurat object, and the top 1,500 highly variable genes were identified using the “FindVariableFeatures” function. After that, we applied the “RunPCA” function of the “Seurat” R package to perform the principal component analysis (PCA) to reduce the dimension of the scRNA-seq data based on the top 1,500 genes. We used JackStraw analysis to identify significant PCs, and we selected the first 15 PCs for cell clustering analysis according to the proportion of variance explained. The “FindNeighbors” and “FindClusters” functions in the “Seurat” package were used for cell clustering analysis. The k-nearest neighbor graph was constructed based on Euclidean distance in PCA using the “FindNeighbors” function to determine the closest neighbors of each cell. Then, t-distributed stochastic neighbor embedding (t-SNE) was performed using the “RunTSNE” function. Cell clustering was demonstrated using t-SNE-1 and t-SNE-2. The “FindAllMarkers” function in the “Seurat” package was used to calculate the differentially expressed genes (DEGs) of each cluster using Wilcoxon–Mann–Whitney tests. To identify the marker genes for each cluster, the cutoff threshold values, adjusted p-value <0.01 and |log2 (fold change)| >1 were used. For cluster annotation, we performed a reference-based annotation using reference data from the Human Primary Cell Atlas (34).

### Construction and Validation of Prognostic Signature Based on NK Cell Marker Genes

A univariate Cox regression analysis was performed to evaluate the prognostic value of NK cell marker genes for OS in TCGA LUAD patients, and genes with p <0.01 were identified as prognostic genes. Next, to minimize overfitting, prognostic genes were assessed by least absolute shrinkage and selection operator (LASSO) Cox proportional hazards regression using the “glmnet” package. LASSO is a popular method for regression with high-dimensional predictors and is broadly applied to the Cox proportional hazard regression model for survival analysis (35). By using the function “cv.glmnet”, 10-fold cross-validation was conducted to select the best model. The tuning parameter λ was chosen by 1 − SE (standard error). We got a list of genes with non-zero beta coefficients. Finally, based on the genes generated by LASSO Cox regression analysis, we used a stepwise multivariate Cox regression analysis to identify the prognostic values of specific gene signatures. The risk model was constructed by a linear combination of the mRNA expression of the genes and the relevant risk coefficient. Based on the median cut-off value, the patients were classified into the low-risk or high-risk groups. To validate the prognostic power of the NKCMGS, the area under the curve (AUC) was calculated using the “survivalROC” package (36). The Kaplan–Meier method was employed for survival analysis, and the log-rank test was used to determine the statistical significance of the differences using the R package “survminer” (37). The predictive ability of the signature was validated using survival analysis and AUC in 6 independent GEO datasets.

### Pathway and Function Enrichment Analysis

We performed Gene Ontology (GO) and Kyoto Encyclopedia of Genes and Genomes (KEGG) analysis by using the R package “clusterProfiler” (38). GO analysis was performed using the enrichGO function of the R package “clusterProfiler” and GO annotations were based on genome-wide annotation packages (org.Hs.eg.db) released by the Bioconductor project (39). KEGG analysis was performed using the enrichKEGG function of the R package “clusterProfiler” and “clusterProfiler” queries the latest online KEGG database through a web API to obtain the pathway data and perform functional analysis. A p-value of < 0.05 was considered significant enrichment.

### Immune Cell Infiltration Analysis and Gene Sets Variation Analysis (GSVA)

The CIBERSORT algorithm, a useful method for obtaining infiltrating characteristics of 22 immune cell types with gene expression profiles (40), was applied to dissect the proportion of immune cell infiltration in high-risk and low-risk groups. A seven-metagene (HCK, IgG, Interferon, LCK, MHC-I, MHC-II, and STAT1) has been extensively used to assess the inflammatory activity in TME (41). Therefore, we conducted GSVA analysis to investigate the associations between the NKCMGS and metagenes of inflammatory activities by using the “GSVA” package (42). Heatmap plots were generated using the “ComplexHeatmap” R package from Bioconductor (43).

### Estimation of Stromal and Immune Scores

The ESTIMATE algorithm was employed to assess levels of stromal and immune cell infiltration using expression profiles by the “estimate” R package (44). Stromal score, immune score, ESTIMATE score, and tumor purity score were calculated using the RNA-sequencing data of the TCGA LUAD cohort and a Wilcoxon t-test was performed to compare these scores between different risk groups.

### Immunotherapy Response Prediction

We first applied PD-L1 expression, tumor mutation burden (TMB), and TCR repertoire to predict the response to immune checkpoint blocking therapy. The PD-L1 mRNA expression of LUAD patients was collected from RNA-sequencing data of the TCGA LUAD cohort. Gene mutation data of LUAD patients were downloaded from the TCGA database and TMB was calculated using “maftools” package (45). TMB was determined as the number of somatic indels and base substitutions per million bases in the coding region of the genome detected, and was calculated as previously described (46). The richness and Shannon diversity indexes were used to characterize the diversity of the TCR repertoire. The richness measures the number of unique TCRs in the sample, while the Shannon diversity index reflects the relative abundance of the different TCRs. The richness values and Shannon diversity index valves of TCR in the TCGA LUAD patients were obtained from the Pan-Cancer Atlas study (47). Additionally, 298 urothelial carcinoma patients with both transcriptomic data and treatment response to immunotherapy from the IMvigor210 cohort were used for speculating the immunotherapy response of the signature.

### Statistical Analysis

Categorized variables between different risk groups were compared by the Wilcoxon t-test. Univariate and multivariate Cox regression analyses were used to investigate the prognostic value of the NKCMGS and different clinicopathological characteristics. P <0.05 was set as a significant threshold. Benjamini–Hochberg was implemented to adjust the P-value for multiple testing using the R function “p.adjust”. For data analysis and generation of figures, R software version 4.1.0 (http://www.R-project.org) was used.

## Results

### Identification of NK Cell Marker Gene Expression Profiles

**Figure 1:**
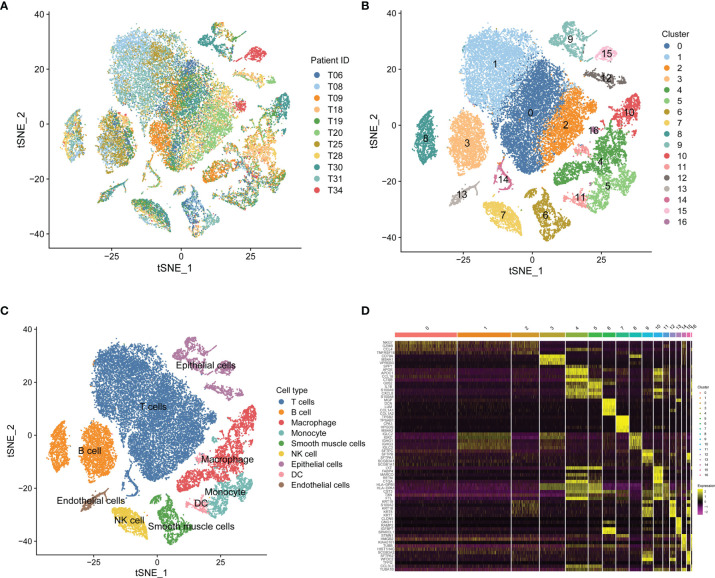
Single-cell RNA-sequencing analysis identifies NK cell marker genes. **(A)** t-SNE plot of 45,149 cells from 11 primary LUAD samples. **(B)** t-SNE plot colored by various cell clusters. **(C)** The cell types identified by marker genes. **(D)** Heatmap showing the top 5 marker genes in each cell cluster.

Based on scRNA-seq data of GSE131907, we obtained gene expression profiles of 45,149 cells from 11 primary LUAD samples for further analysis (**Figure 1A**). We conducted PCA using the top 1,500 variable genes to reduce the dimensionality, and 17 cell clusters were then identified (**Figure 1B**). Subsequently, the cell identity of each cluster was annotated using a reference dataset from the Human Primary Cell Atlas, and cells in cluster 7 were defined as NK cells (**Figure 1C**). This cluster was also found to have distinct gene expression profiles, with 189 genes differentially expressed between the 17 clusters (**Figure 1D**), which were identified as LUAD-related NK cell marker genes (**Supplementary Table 2**). The functional enrichment, including GO and KEGG analysis, showed that the NK cell marker genes were mostly related to immune features, such as positive regulation of leukocyte activation, MHC protein complex, antigen binding, and hematopoietic cell lineage (**Supplementary Figure 1**).

### Establishment of the Seven-Gene Prognostic Signature Based on NK Cell Marker Genes

**Figure 2:**
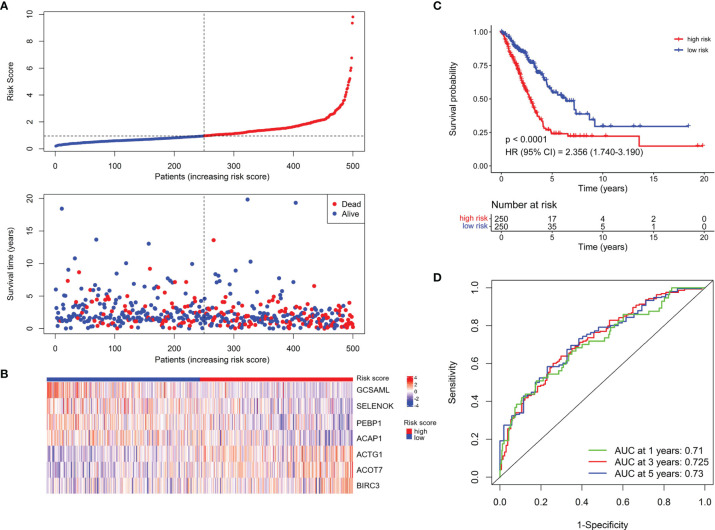
Establishment of the NKCMGS in the TCGA LUAD cohort. **(A)** The distribution of risk score and survival status. **(B)** Heatmap showed the expression characteristics of the identified 7 NK cell marker genes. **(C)** Kaplan–Meier curves of survival analysis compared the overall survival of LUAD patients between high-risk and low-risk groups. **(D)** ROC curves of the NKCMGS for predicting the risk of death at 1, 3, and 5 years.

To construct a prognostic signature based on the 189 NK cell marker genes, we first used the TCGA LUAD cohort as the training set to perform a univariate Cox regression analysis, and 25 NK cell marker genes were significantly related to OS (**Supplementary Table 3**). Next, LASSO Cox regression analysis with one standard error (SE) and 10-fold cross-validation was conducted on the 25 NK cell marker genes, and 16 genes were screened out (HPGDS, CTSG, SLC18A2, GCSAML, ADRB2, ACTG1, ACOT7, CLIC1, SELENOK, PEBP1, BEX4, BIRC3, DDIT4, TRBC1, ACAP1, and S100A10) for further analysis (**Supplementary Figures 2A, B**). Finally, we used stepwise multivariate Cox regression analysis to optimize the prognostic signature to only include the 7 most predictive genes: Risk score = (−0.614 × GCSAML expression) + (1.893 × ACTG1 expression) + (1.022 × ACOT7 expression) + (−1.715 × SELENOK expression) + (−1.840 × PEBP1 expression) + (1.077 × BIRC3 expression) + (−1.180 × ACAP1 expression) (**Supplementary Figure 2C**). The relative expression of the 7 marker genes in various clusters is shown in **Supplementary Figure 3**, which indicates the specificity of the expression of the signature genes. The median risk score was 0.956 by ranking the risk score from high to low, which was used to classify patients into low-risk (n = 250) and high-risk (n = 250) groups. **Figure 2A** exhibited the distribution of risk scores and survival status, which suggested more deaths in the high-risk group. **Figure 2B** shows the expression details of the 7 NK cell marker genes.

Kaplan–Meier analysis demonstrated that patients with high-risk scores had significantly inferior OS than patients with low-risk scores (**Figure 2C**). To assess the predictive accuracy of the risk model, time-dependent area under the ROC curves for OS was calculated, and the 1-, 3-, and 5-year AUC values were 0.710, 0.725, and 0.730, respectively (**Figure 2D**). The performance of the model was then evaluated using a ten-fold cross-validation procedure, and the 1-, 3-, and 5-year mean AUC values were 0.669, 0.674, and 0.652, respectively.

### Validation of the NKCMGS in Different Clinical Subgroups

The predictive value of NKCMG was first assessed in TCGA LUAD patients with different genders, ages, smoking histories, and tumor stages. The results revealed that high-risk score was significantly correlated with an inferior prognosis in the male (*P* = 1.5 × 10^−4^, **Supplementary Figure 4A**), female (*P =* 3.563 × 10^−5^, **Supplementary Figure 4B**), young (*P* = 7.8 × 10^−4^, **Supplementary Figure 4C**) or old (*P =* 1.306 × 10^−6^, **Supplementary Figure 4D**), non-smoker (*P* = 0.016, **Supplementary Figure 4E**), smoker (*P =* 4.465 ×10^−6^, **Supplementary Figure 4F**), early stage (*P =*1.709 × 10^−5^, **Supplementary Figure 4G**) or advanced stage (*P* = 0.0094, **Supplementary Figure 4H**) LUAD patients. Next, we further evaluated the predictive performance of NKCMGS in the TCGA LUAD patients stratified by different molecular characteristics, including EGFR, KRAS, and TP53 mutations. Similarly, we observed that the NKCMGS showed robust predictive power in the EGFR wild-type (WT) (*P =* 9.923 × 10^−7^, **Supplementary Figure 5A**), EGFR mutation (MUT) (*P* = 0.032, **Supplementary Figure 5B**), KRAS WT (*P =* 1.23 × 10^−6^, **Supplementary Figure 5C**), KRAS MUT (*P* = 0.012, **Supplementary Figure 5D**), TP53 WT (*P* = 6.9 × 10^−4^, **Supplementary Figure 5E**) or TP53 MUT (*P =* 2.377 × 10^−5^, **Supplementary Figure 5F**) subgroup.

### External Validation of the Robustness of the NKCMGS in Six Independent Cohorts

**Figure 3:**
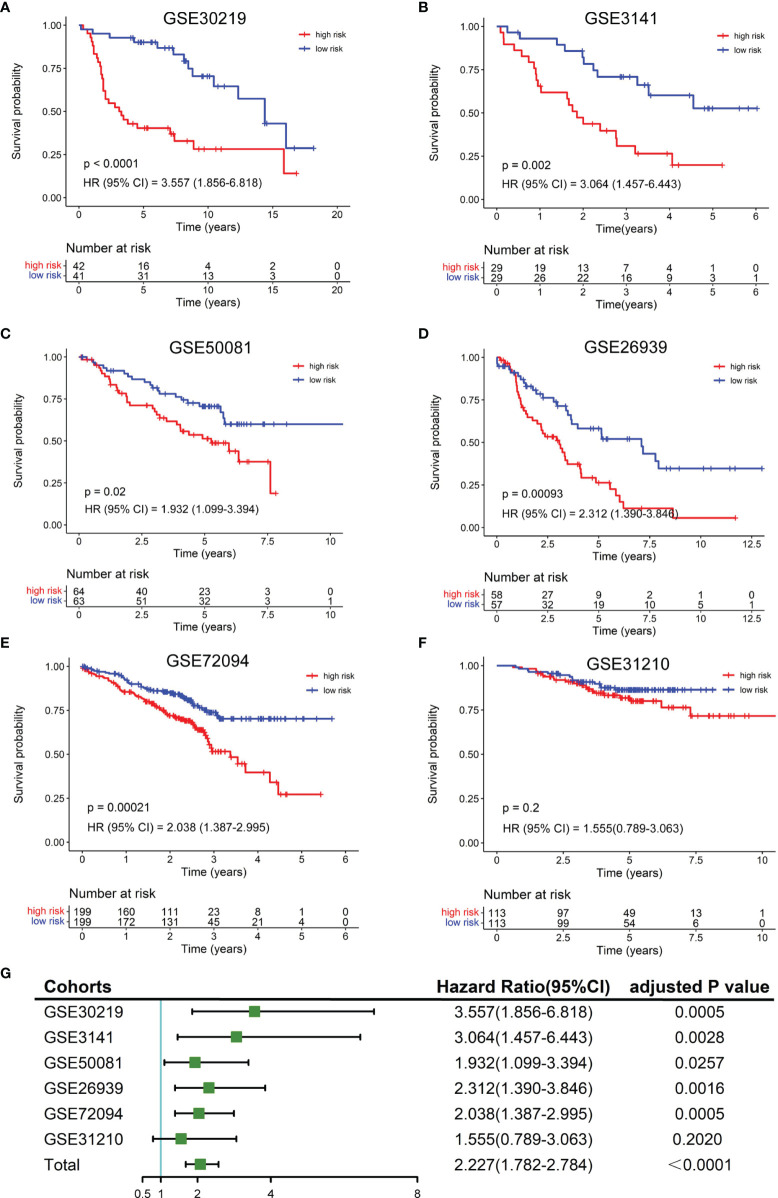
Validation of the NKCMGS in six independent GEO cohorts. **(A)** GSE31210 (n = 226). **(B)** GSE30219 (n = 83). **(C)** GSE37745 (n = 106). **(D)** GSE50081 (n = 127). **(E)** GSE26939 (n = 115). **(F)** GSE42127 (n = 133). **(G)** A meta-analysis of six GEO cohorts and the P-value was adjusted for 7 hypothesis tests.

To validate the robustness of the NKCMGS, we included 6 independent GEO cohorts in this study, and the clinical features of these 6 GEO cohorts are shown in **Table 1**. We used the same formula to calculate the risk score of each patient in 6 GEO cohorts. Patients were sorted into the high-risk and low-risk groups in each cohort by the median risk score. Kaplan–Meier analysis demonstrated that the high-risk group had inferior prognosis than the low-risk group in all 6 GEO cohorts, namely, GSE30219 (**Figure 3A**, HR: 3.557, 95% CI: 1.856–6.818, *P* = 1.32 × 10^−4^), GSE3141 (**Figure 3B**, HR: 3.064, 95% CI: 1.457–6.443, *P* = 0.002), GSE50081 (**Figure 3C**, HR: 1.932, 95% CI: 1.099–3.394, *P* = 0.02), GSE26939 (**Figure 3D**, HR: 2.312, 95% CI: 1.390–3.846, *P* = 9.3 × 10^−4^), GSE72094 (**Figure 3E**, HR: 2.038, 95% CI: 1.387–2.995, *P* = 2.1 × 10^−4^), and GSE31210 (**Figure 3F**, HR: 1.555, 95% CI: 0.789–3.063, *P* = 0.2). The ROC curves of the risk score in the 6 validation cohorts also showed good performance (**Supplementary Figure 6**). Additionally, a prognostic meta-analysis was performed by R package “meta” using the random-effects model to evaluate the integrated predictive value of NKCMGS in these 6 cohorts (48). The results of the meta-analysis indicated that NKCMGS was a significant prognostic indicator for patients with LUAD (HR: 2.227, 95% CI: 1.782–2.784, *P* = 1.96 × 10^−12^) (**Figure 3G**).

**Table 1:** Clinical characteristics of lung adenocarcinoma from multiple cohorts.

Variables	TCGA *N* = 500	GSE30219 *N* = 83	GSE3141 *N* = 58	GSE50081 *N* = 127	GSE26939 *N* = 115	GSE72094 *N* = 398	GSE31210 *N* = 226
Age (year)							
Median	66	60	–	70	65	70	61
Range	33–88	44–84	–	40–86	41–90	64–77	30–76
Gender							
Male	230	65	–	65	53	176	105
Female	270	18	–	62	62	222	121
Smoking							
Yes	415	–	–	92	100	300	111
No	71	–	–	23	12	31	115
NA	14	–	–	12	3	67	0
TNM stage							
I and II	387	83	–	127	71	321	226
III and IV	105	0	–	0	16	72	0
NA	8	0	–	0	28	5	0
OS Status							
Alive	318	40	26	76	49	285	191
Death	182	43	32	51	66	113	35

NA, not available; OS, overall survival.

### Independent Prognostic Role of the NKCMGS for Patients With LUAD

To further investigate whether the risk score can independently affect the prognosis of LUAD, we conducted univariate and multivariate Cox regression analysis using clinical features, molecular factors, and the risk score in the TCGA LUAD patients. As expected, the results of multivariate Cox regression analysis proved that the risk score was an independent prognostic factor (HR: 1.889, 95% CI: 1.373–2.599, *P* = 9.37 × 10^−5^) (**Table 2**). Meanwhile, we performed a LASSO Cox regression analysis with the risk score and all these clinical features to select the most predictive variables. The results demonstrated that the risk score and tumor stage were the best predictive factors for the prognosis.

**Table 2:** Univariable and multivariable Cox regression analysis of the NK cell marker genes signature in TCGA LUAD cohort.

Characteristics	Univariable analysis			Multivariable analysis		
	HR	95% CI	*P*-Value	HR	95% CI	*P*-Value
**Age**						
≤60	1.0 (ref)					
>60	1.217	0.906–1.635	0.192			
**Gender**						
Female	1.0 (ref)					
Male	1.049	0.784–1.405	0.747			
**Smoking history**						
No	1.0 (ref)					
Yes	0.881	0.583–1.330	0.546			
**T stage**						
T1 + T2	1.0 (ref)			1.0 (ref)		
T3 + T4	2.298	1.568–3.366	<0.001	1.646	1.077–2.515	0.021
**Lymphatic metastasis**						
No	1.0 (ref)			1.0 (ref)		
Yes	2.579	1.918–3.469	<0.001	1.903	1.326–2.733	<0.001
**TNM stage**						
I + II	1.0 (ref)			1.0 (ref)		
III + V	2.584	1.893–3.527	<0.001	1.337	0.885–2.022	0.168
**EGFR mutation**						
No	1.0 (ref)					
Yes	1.332	0.872–2.035	0.185			
**KRAS mutation**						
No	1.0 (ref)					
Yes	1.068	0.764–1.494	0.698			
**TP53 mutation**						
No	1.0 (ref)			1.0 (ref)		
Yes	1.413	1.054–1.893	0.021	1.152	0.851–1.561	0.359
**Risk score**						
Low	1.0 (ref)			1.0 (ref)		
High	2.356	1.740–3.190	<0.001	1.889	1.373–2.599	<0.001

HR, hazard ratio; CI, confidence interval; ref, reference category.

### Functional Enrichment Analysis of the NKCMGS Related Genes

To elucidate the potential mechanism of the excellent predictive capability of NKCMGS, we further investigated biological pathways related to NKCMGS. Firstly, the correlation analysis was performed using the TCGA LUAD dataset to identify the genes that were closely correlated with the risk score (Pearson |R| >0.4, *P <*0.05). As shown in **Supplementary Figure 7A**, 100 positively correlated genes and 24 negatively correlated genes were filtered out (**Supplementary Table 4**). Subsequently, we performed GO and KEGG enrichment analyses using the “ClusterProfiler” package for these selected genes. GO analysis revealed that these genes were mainly implicated in the biological processes of mitotic division, namely, chromosome segregation, mitotic nuclear division, and the G2/M transition of the mitotic cell cycle (**Supplementary Figure 7B**). KEGG analysis also verified that these genes were closely involved in the cell cycle pathway (**Supplementary Figure 7C**).

### The NKCMGS Was Associated With the Immune Cell Infiltration of the TME

**Figure 4:**
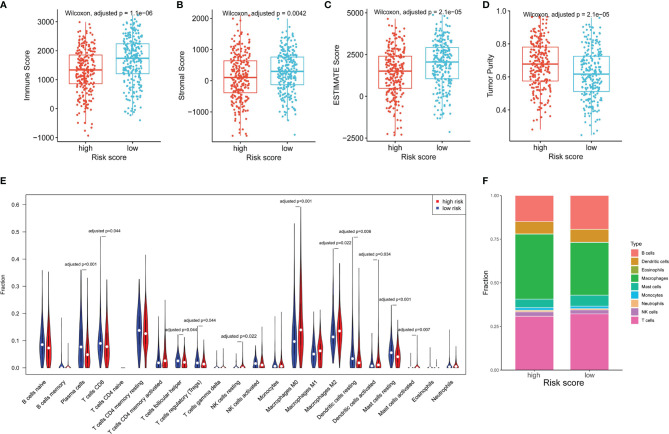
The association between the NKCMGS and the immune cell infiltration in the TME. Differences among immune score **(A)**, stromal score **(B)**, ESTIMATE score **(C)**, and tumor purity **(D)** between high-risk and low-risk groups. The P-value was adjusted for 4 hypothesis tests. **(E)** The comparison of immune cells infiltration level of 22 immune cell types between high-risk and low-risk groups. **(F)** The fractions of different immune cells between high- and low-risk groups. The P-*value was adjusted for 22 hypothesis tests.

As NK cells play a vital role in anti-tumor immunity, we explored the relationship of the NKCMGS with immune cell infiltration in LUAD patients. By using the ESTIMATE algorithm, we found that high-risk patients had lower immune score, stromal score, ESTIMATE score, and higher tumor purity than low-risk patients (**Figures 4A–D**), which suggested that the risk score was negatively correlated with the level of immune cell infiltration. Subsequently, we applied the CIBERSORT algorithm to estimate the infiltration level of different types of immune cells in the TME. The CIBERSORT analysis revealed that high-risk patients had a higher fraction of resting NK cells, M0 macrophages, M2 macrophages, activated dendritic cells, and activated mast cells, but had a lower fraction of plasma cells, CD8^+^ T cells, follicular helper T cells, regulatory T cells, resting dendritic cells, and resting mast cells (**Figure 4E**). In **Figure 4F**, the fractions of different immune cells between high- and low-risk groups are shown. We further conducted a correlation analysis between the risk score and immune cell infiltration, which showed that the risk score was positively related to macrophages and neutrophils but was negatively related to T, B, and mast cells (**Supplementary Figure 8**).

### Inflammatory and Immune Profiles of the NKCMGS

To figure out the relationship between NKCMGS and inflammatory activities, we explored the associations between the NKCMGS and 7 clusters of metagenes (HCK, LCK, IgG, Interferon, MHC-I, MHC-II, and STAT1), representing various inflammatory and immune activities as previously reported (41). **Supplementary Figure 9A** shows the expression details of these metagenes in the TCGA LUAD dataset. Next, we used Gene Sets Variation Analysis (GSVA) to calculate the expression of 7 gene clusters and the correlation between the NKCMGS and each cluster of metagenes is shown in **Supplementary Figure 9B**. The results showed that the risk score was negatively correlated with HCK, IgG, LCK, MHC-I, and MHC-II.

### The NKCMGS Could Predict Immunotherapy Benefits in LUAD Patients

**Figure 5:**
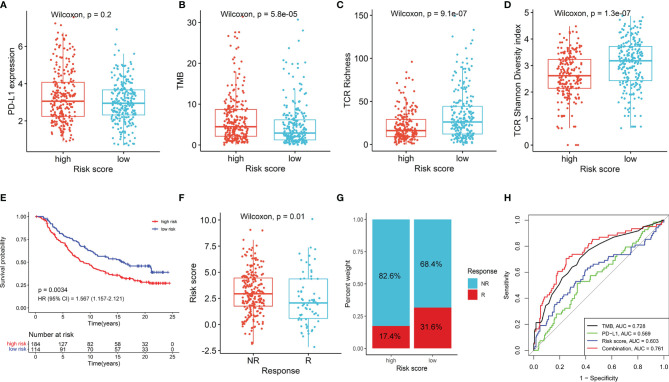
The role of NKCMGS in predicting immunotherapeutic benefit. The comparison of PD-L1 expression **(A)**, tumor mutation burden (TMB) **(B)**, TCR richness **(C)**, TCR diversity **(D)** between high-risk and the low-risk patients. **(E)** Kaplan–Meier curves for patients with high-risk and low-risk scores in the IMvigor210 cohort. **(F)** The comparison of risk scores in groups with different anti-PD-L1 treatment response status in the IMvigor210 cohort. NR represents progressive disease (PD)/stable disease (SD); R represents complete response (CR)/partial response (PR). **(G)** Treatment response rates of anti-PD-L1 immunotherapy in high- and low-risk groups in the IMvigor210 cohort (*P* = 0.005). NR represents SD/PD; R represents CR/PR. **(H)** ROC curves evaluating the predictive accuracy of the TMB, PD-L1, risk score, and combination of TMB, PD-L1, and risk score in the IMvigor210 cohort.

Given the important roles in anti-tumor immunity of NK cells and the promising treatment efficacy of NK cell-based immunotherapy, we explored whether the NKCMGS could predict responses of LUAD patients to immune checkpoint inhibitors. First, we analyzed the relationship between the NKCMGS and widely recognized immunotherapy biomarkers (PD-L1 expression and TMB) in the TCGA LUAD cohort. The results indicated that PD-L1 expression exhibited no significant difference between the low-risk and high-risk patients, but low-risk patients harbored a significantly lower TMB than high-risk patients (**Figures 5A, B**). In previous studies, the T-cell receptor (TCR) is in charge of the recognition of antigens presented by the MHC, and the repertoire analysis of TCR has been demonstrated as a useful biomarker for stratification and monitoring of patients on immunotherapy (49–51). Subsequently, we analyzed the TCR repertoire and found that the TCR richness and diversity of low-risk patients was significantly higher than that of high-risk LUAD patients (**Figures 5C, D**). Finally, to further explore the value of NKCMGS in predicting the immunotherapy response, 298 patients from the IMvigor210 cohort who received anti-PD-L1 treatment were enrolled in this study for analysis. Kaplan–Meier analysis showed an inferior survival rate for high-risk patients after immunotherapy (**Figure 5E**). Lower risk scores were associated with an objective response to anti-PD-L1 treatment (Wilcoxon test, *P* = 0.01; **Figure 5F**). The objective response rate of anti-PD-L1 treatment was significantly elevated in the low-risk group (two-sided chi-square test, *P* = 0.005; **Figure 5G**). The ROC curves showed that the combination of TMB, PD-L1, and risk score models could predict anti-PD-L1 response with 76.1% accuracy, which was superior to that of TMB (AUC = 0.728), PD-L1 (AUC = 0.569), or risk score (AUC = 0.603) alone (**Figure 5H**). Collectively, these findings indicate that patients with a low-risk score are more likely to benefit from immunotherapy, and the NKCMGS may be a useful biomarker to identify LUAD patients who may benefit from immunotherapy.

## Discussion

With the rapid development of scRNA-seq technologies, researchers are increasingly exploring molecular characteristics of tumor-infiltrating immune cells in the TME. However, most current studies have focused on adaptive immune cells, and the roles of innate immune cells have not yet received enough attention, which may markedly affect the prognosis and treatment response, especially with immunotherapy. The abundance of tumor-infiltrating NK cells is tightly associated with the prognosis of patients with various solid tumors (18–21). Recently, Cursons et al. developed a transcriptional signature based on NK cell marker genes to evaluate nature killer (NK) cell infiltration in the TME, and an increased NK score significantly stratified patients with superior prognosis in metastatic cutaneous melanoma (21). Inspired by this research, we attempted to explore the NK cell marker genes of LUAD through scRNA-seq analysis in our study. A novel prognostic prediction signature based on NK cell marker genes (NKCMGS) was developed for LUAD patients in the TCGA database and well verified in 6 independent cohorts from the GEO dataset. Low-risk scores of NKCMGS were closely correlated with abundant infiltration of immune cells and a high level of TCR richness and diversity. Furthermore, we discovered that the immunotherapy response rate of patients with low-risk scores was dramatically higher than that of patients with high-risk scores, indicating that immune checkpoint blockade therapy is more appropriate for low-risk patients.

In this study, NKCMGS was composed of 7 NK cell marker genes (GCSAML, ACTG1, ACOT7, SELENOK, PEBP1, BIRC3, and ACAP1), most of which are correlated with the prognosis of LUAD patients or the activity of NK cells. ACTG1 encodes γ-actin, which is a component of the cytoskeleton. Increased expression of ACTG1 was linked to the enhanced ability of cell migration in lung adenocarcinoma (52) and upregulated expression of ACTG1 was also markedly associated with poor prognosis in patients with lung cancer (53). As one of the acyl-CoA metabolic enzymes, ACOT7 was implicated in the progression of LUAD by regulating the cell cycle through the p53/p21 signaling pathway (54), which is consistent with our findings that the signature was closely related to the cell cycle pathway. ACOT7 expression levels were found to be high in LUAD and were linked to impaired prognosis (54). SELENOK encodes a membrane selenoprotein (SelK), which is expressed abundantly in NK cells and is involved in regulating the function of NK cells (55, 56). SELENOK may modulate cell proliferation and migration through regulating Ca^+^ flux (56), and the expression of SELENOK was also associated with a poor prognosis in LUAD (57). Moreover, PEBP1, also known as Raf kinase inhibitory protein (RKIP), was downregulated in LUAD tissues compared with normal adjacent tissues (58). Low PEBP1 expression led to reduced survival in LUAD, and *in vitro* experiments demonstrated that upregulation of PEBP1 expression can suppress the proliferation and invasion of LUAD cells, which indicates that PEBP1 may act as a tumor suppressor gene (58). BIRC3 is a hallmark of tumor-infiltrating NK cells, and upregulation of BIRC3 can inhibit NK cell activity (59). Besides, BIRC3 expression was dramatically higher in LUAD tissues, and higher BIRC3 expression was correlated with a poorer prognosis (60). These reports indicated that genes identified in the NKCMGS might provide potential targets for experimental design in the laboratory to illuminate molecular mechanisms in LUAD.

In this study, the NKCMGS prognostic signature proved to be a powerful predictive tool for the prognosis of patients in both training and validation cohorts. The excellent predictive ability of the NKCMGS inspired us to investigate the potential underlying mechanism. We first performed GO and KEGG analyses to explore the enriched pathways for genes closely related to the NKCMGS and found that these correlated genes were mostly enriched in the biological processes of cell division and cell cycle pathway. Hence, the inferior prognosis of patients with high-risk scores may be partly attributed to the abnormal regulation of the cell cycle, which is intimately linked to tumor proliferation and progression (61). Besides, tumor-infiltrating immune cells in the TME play a vital role in tumor development and significantly affect prognosis (62). We then compared the abundance of immune cell infiltration between high-risk and low-risk groups by ESTIMATE and CIBERSORT algorithms. The results revealed that high-risk tumors had a lower infiltration level of immune cells, especially T and B cells, which suggested that tumors with a high-risk score were characterized as so-called “cold tumors” with decreased anti-tumor activity (63). The low level of immune cell infiltration can promote tumor cell escape from immune surveillance and facilitate tumor progression, which may partly account for the significantly decreased survival of high-risk LUAD patients.

Furthermore, the NKCMGS was evaluated in relation to immune and inflammatory activities by analyzing immune-related metagenes, and the risk score was found to correlate negatively with HCK, IgG, LCK, MHC-I, and MHC-II clusters. HCK is pivotal in innate immunity by regulating the phagocytosis of neutrophils and macrophages (64). LCK, a Src-related protein linked to CD8 and CD4 molecules, is required for the maturation and stimulation of T cells (65). MHC-I and MHC-II are closely associated with the function of antigen-presentation and tumor cells can escape T-cell killing by losing the expression of MHC-I and MHC-II (66). Therefore, high-risk patients showed an immunosuppressive microenvironment, which may be partly responsible for the significantly inferior prognosis. Collectively, according to all the findings above, we inferred that the potential mechanism of the powerful predictive ability of the NKCMGS may lie in the dysregulation of the cell cycle and immunosuppressive microenvironment.

The discrepancy in immune cell infiltration and inflammatory activities between different risk groups prompted us to explore the value of the NKCMGS in predicting immunotherapy response. We first analyzed the association between the NKCMGS and the well-recognized biomarkers, including PD-L1 expression TMB. The results revealed that PD-L1 expression showed no significant difference between high-risk and low-risk patients, but low-risk patients harbored a significantly lower TMB, which indicated the low immunogenicity of low-risk tumors. TCR is a unique molecule on the T-cell surface that recognizes antigens presented by MHC. Several studies have recently used high-throughput TCR sequencing to analyze the characteristics of T-cell repertoires in patients with diverse cancer types, demonstrating that TCR repertoires could act as a potent tool to predict immunotherapy response (49–51). We evaluated the richness and diversity of the TCR repertoire and discovered that low-risk patients were associated with a higher level of TCR richness and diversity, which reflected higher functionality of T cells in recognizing antigens and killing tumor cells in low-risk LUAD patients. The success of immune checkpoint blockade therapy was associated with many factors, namely, the immunogenicity of the tumor, the abundance and functionality of tumor-infiltrating T cells, and the expression of immune checkpoints. In this study, although low-risk tumors had lower immunogenicity, the abundance and functionality of tumor-infiltrating T cells in low-risk groups was dramatically elevated compared with that in high-risk groups. Therefore, an immunotherapy cohort was needed to verify the predictive value of the NKCMGS. By using an immunotherapy cohort (Imvigor210), we explored the ability of NKCMGS to predict immunotherapeutic efficacy and observed that low-risk patients were more sensitive to anti-PD-L1 therapy response, which demonstrated that the impact of the abundance and functionality of tumor-infiltrating T cells on immunotherapy response is more important than tumor immunogenicity. Taken together, low-risk patients were more likely to benefit from immunotherapy. With further validation, NKCMGS might act as a reliable biomarker for predicting immunotherapy response.

Despite the promising findings obtained, this study has several limitations. First, the expression and prognostic role of the genes in NKCMGS at protein-level warrant further investigation. Second, the candidate genes involved in our study were restricted to the NK cell marker genes, and the tumor immune microenvironment is highly spatially heterogeneous. Hence, the prognosis-predicting ability of the signature was limited. Lastly, all the mechanistic analysis in our study was descriptive. Future research must explore the underlying mechanism between the expression of genes in NKCMGS and the prognosis of LUAD.

In conclusion, a seven-gene signature based on NK cell marker genes was identified and validated to have powerful performance to predict prognosis and immunotherapy response in LUAD patients. It might serve as a prognostic biomarker for clinical decision-making regarding individualized prediction and facilitate the selection of appropriate patients who can benefit from immunotherapy.

## Data Availability Statement

The results shown here are in whole based on data generated by the TCGA Research Network: https://www.cancer.gov/tcga, GEO database: https://www.ncbi.nlm.nih.gov/geo/ under the accession numbers GSE131907, GSE30219, GSE3141, GSE50081, GSE26939, GSE72094, GSE31210, and IMvigor210 cohort: http://research-pub.gene.com/IMvigor210CoreBiologies.

## Author Contributions

SG and JH supervised the project and designed this study. WL organized the public data and prepared all the figures and tables. LG conducted the data analysis. PS drafted the manuscript. JY revised the manuscript. All authors listed have made a substantial, direct, and intellectual contribution to the work and approved it for publication.

## Funding

The study was supported by the National Key R&D Program of China (2021YFC2500900); the National Key R&D Program of China (2020AAA0109500); the R&D Program of Beijing Municipal Education commission (KJZD20191002302); the Beijing Municipal Science & Technology Commission (Z191100006619119); the Beijing Hope Run Special Fund of Cancer Foundation of China (LC2019A11); and the Beijing Nova Program of Science and Technology (Z191100001119095).

## Conflict of Interest

The authors declare that the research was conducted in the absence of any commercial or financial relationships that could be construed as a potential conflict of interest.

## Publisher’s Note

All claims expressed in this article are solely those of the authors and do not necessarily represent those of their affiliated organizations, or those of the publisher, the editors and the reviewers. Any product that may be evaluated in this article, or claim that may be made by its manufacturer, is not guaranteed or endorsed by the publisher.

## References

[1] Sung H Ferlay J Siegel RL Laversanne M Soerjomataram I Jemal A. Global Cancer Statistics 2020: GLOBOCAN Estimates of Incidence and Mortality Worldwide for 36 Cancers in 185 Countries. CA Cancer J Clin (2021) 71(3):209–49. doi: 10.3322/caac.21660 33538338

[2] Testa U Castelli G Pelosi E. Lung Cancers: Molecular Characterization, Clonal Heterogeneity and Evolution, and Cancer Stem Cells. Cancers (Basel) (2018) 10(8):248. doi: 10.3390/cancers10080248 PMC6116004 30060526

[3] Little AG Gay EG Gaspar LE Stewart AK. National Survey of Non-Small Cell Lung Cancer in the United States: Epidemiology, Pathology and Patterns of Care. Lung Cancer (2007) 57(3):253–60. doi: 10.1016/j.lungcan.2007.03.012 17451842

[4] Chang JT Lee YM Huang RS. The Impact of the Cancer Genome Atlas on Lung Cancer. Transl Res (2015) 166(6):568–85. doi: 10.1016/j.trsl.2015.08.001 PMC4656061 26318634

[5] Imielinski M Berger AH Hammerman PS Hernandez B Pugh TJ Hodis E. Mapping the Hallmarks of Lung Adenocarcinoma With Massively Parallel Sequencing. Cell (2012) 150(6):1107–20. doi: 10.1016/j.cell.2012.08.029 PMC3557932 22980975

[6] Topalian SL Hodi FS Brahmer JR Gettinger SN Smith DC McDermott DF. Safety, Activity, and Immune Correlates of Anti-PD-1 Antibody in Cancer. N Engl J Med (2012) 366(26):2443–54. doi: 10.1056/NEJMoa1200690 PMC3544539 22658127

[7] Sharma P Allison JP. The Future of Immune Checkpoint Therapy. Science (2015) 348(6230):56–61. doi: 10.1126/science.aaa8172 25838373

[8] Gibney GT Weiner LM Atkins MB. Predictive Biomarkers for Checkpoint Inhibitor-Based Immunotherapy. Lancet Oncol (2016) 17(12):e542–e51. doi: 10.1016/S1470-2045(16)30406-5 PMC5702534 27924752

[9] Sharma P Hu-Lieskovan S Wargo JA Ribas A. Primary, Adaptive, and Acquired Resistance to Cancer Immunotherapy. Cell (2017) 168(4):707–23. doi: 10.1016/j.cell.2017.01.017 PMC5391692 28187290

[10] Hanahan D Coussens LM. Accessories to the Crime: Functions of Cells Recruited to the Tumor Microenvironment. Cancer Cell (2012) 21(3):309–22. doi: 10.1016/j.ccr.2012.02.022 22439926

[11] Hanahan D Weinberg RA. The Hallmarks of Cancer. Cell (2000) 100(1):57–70. doi: 10.1016/S0092-8674(00)81683-9 10647931

[12] Binnewies M Roberts EW Kersten K Chan V Fearon DF Merad M. Understanding the Tumor Immune Microenvironment (TIME) for Effective Therapy. Nat Med (2018) 24(5):541–50. doi: 10.1038/s41591-018-0014-x PMC5998822 29686425

[13] Moretta L. NK Cell-Mediated Immune Response Against Cancer. Surg Oncol (2007) 16 Suppl 1:S3–5. doi: 10.1016/j.suronc.2007.10.043 18032029

[14] Vivier E Ugolini S Blaise D Chabannon C Brossay L. Targeting Natural Killer Cells and Natural Killer T Cells in Cancer. Nat Rev Immunol (2012) 12(4):239–52. doi: 10.1038/nri3174 PMC5161343 22437937

[15] Schmidt L Eskiocak B Kohn R Dang C Joshi NS DuPage M. Enhanced Adaptive Immune Responses in Lung Adenocarcinoma Through Natural Killer Cell Stimulation. Proc Natl Acad Sci USA (2019) 116(35):17460–9. doi: 10.1073/pnas.1904253116 PMC6717259 31409707

[16] Lopez-Soto A Gonzalez S Smyth MJ Galluzzi L. Control of Metastasis by NK Cells. Cancer Cell (2017) 32(2):135–54. doi: 10.1016/j.ccell.2017.06.009 28810142

[17] Imai K Matsuyama S Miyake S Suga K Nakachi K. Natural Cytotoxic Activity of Peripheral-Blood Lymphocytes and Cancer Incidence: An 11-Year Follow-Up Study of a General Population. Lancet (2000) 356(9244):1795–9. doi: 10.1016/S0140-6736(00)03231-1 11117911

[18] Ishigami S Natsugoe S Tokuda K Nakajo A Xiangming C Iwashige H. Clinical Impact of Intratumoral Natural Killer Cell and Dendritic Cell Infiltration in Gastric Cancer. Cancer Lett (2000) 159(1):103–8. doi: 10.1016/S0304-3835(00)00542-5 10974412

[19] Coca S Perez-Piqueras J Martinez D Colmenarejo A Saez MA Vallejo C. The Prognostic Significance of Intratumoral Natural Killer Cells in Patients With Colorectal Carcinoma. Cancer (1997) 79(12):2320–8. doi: 10.1002/(SICI)1097-0142(19970615)79:12<2320::AID-CNCR5>3.0.CO;2-P 9191519

[20] Villegas FR Coca S Villarrubia VG Jimenez R Chillon MJ Jareno J. Prognostic Significance of Tumor Infiltrating Natural Killer Cells Subset CD57 in Patients With Squamous Cell Lung Cancer. Lung Cancer (2002) 35(1):23–8. doi: 10.1016/S0169-5002(01)00292-6 11750709

[21] Cursons J Souza-Fonseca-Guimaraes F Foroutan M Anderson A Hollande F Hediyeh-Zadeh S. A Gene Signature Predicting Natural Killer Cell Infiltration and Improved Survival in Melanoma Patients. Cancer Immunol Res (2019) 7(7):1162–74. doi: 10.1158/2326-6066.CIR-18-0500 31088844

[22] Ascierto ML Bozzano F Bedognetti D Marras F Schechterly C Matsuura K. Inherent Transcriptional Signatures of NK Cells Are Associated With Response to IFNalpha + Rivabirin Therapy in Patients With Hepatitis C Virus. J Transl Med (2015) 13:77. doi: 10.1186/s12967-015-0428-x 25849716 PMC4353456

[23] Costanzo MC Kim D Creegan M Lal KG Ake JA Currier JR. Transcriptomic Signatures of NK Cells Suggest Impaired Responsiveness in HIV-1 Infection and Increased Activity Post-Vaccination. Nat Commun (2018) 9(1):1212. doi: 10.1038/s41467-018-03618-w 29572470 PMC5865158

[24] Melaiu O Chierici M Lucarini V Jurman G Conti LA De Vito R. Cellular and Gene Signatures of Tumor-Infiltrating Dendritic Cells and Natural-Killer Cells Predict Prognosis of Neuroblastoma. Nat Commun (2020) 11(1):5992. doi: 10.1038/s41467-020-19781-y 33239635 PMC7689423

[25] Wu M Mei F Liu W Jiang J. Comprehensive Characterization of Tumor Infiltrating Natural Killer Cells and Clinical Significance in Hepatocellular Carcinoma Based on Gene Expression Profiles. BioMed Pharmacother (2020) 121:109637. doi: 10.1016/j.biopha.2019.109637 31810126

[26] Sun Y Sedgwick AJ Khan MA Palarasah Y Mangiola S Barrow AD. A Transcriptional Signature of IL-2 Expanded Natural Killer Cells Predicts More Favorable Prognosis in Bladder Cancer. Front Immunol (2021) 12:724107. doi: 10.3389/fimmu.2021.724107 34858395 PMC8631443

[27] Sun Y Sedgwick AJ Palarasah Y Mangiola S Barrow AD. A Transcriptional Signature of PDGF-DD Activated Natural Killer Cells Predicts More Favorable Prognosis in Low-Grade Glioma. Front Immunol (2021) 12:668391. doi: 10.3389/fimmu.2021.668391 34539622 PMC8444979

[28] Chen H Ye F Guo G. Revolutionizing Immunology With Single-Cell RNA Sequencing. Cell Mol Immunol (2019) 16(3):242–9. doi: 10.1038/s41423-019-0214-4 PMC6460502 30796351

[29] Liang L Yu J Li J Li N Liu J Xiu L. Integration of scRNA-Seq and Bulk RNA-Seq to Analyse the Heterogeneity of Ovarian Cancer Immune Cells and Establish a Molecular Risk Model. Front Oncol (2021) 11:711020. doi: 10.3389/fonc.2021.711020 34621670 PMC8490743

[30] Li Y Zhao X Liu Q Liu Y. Bioinformatics Reveal Macrophages Marker Genes Signature in Breast Cancer to Predict Prognosis. Ann Med (2021) 53(1):1019–31. doi: 10.1080/07853890.2021.1914343 PMC8253219 34187256

[31] Wagner GP Kin K Lynch VJ. Measurement of mRNA Abundance Using RNA-Seq Data: RPKM Measure Is Inconsistent Among Samples. Theory Biosci (2012) 131(4):281–5. doi: 10.1007/s12064-012-0162-3 22872506

[32] Mariathasan S Turley SJ Nickles D Castiglioni A Yuen K Wang Y. TGFbeta Attenuates Tumour Response to PD-L1 Blockade by Contributing to Exclusion of T Cells. Nature (2018) 554(7693):544–8. doi: 10.1038/nature25501 PMC6028240 29443960

[33] Aran D Looney AP Liu L Wu E Fong V Hsu A. Reference-Based Analysis of Lung Single-Cell Sequencing Reveals a Transitional Profibrotic Macrophage. Nat Immunol (2019) 20(2):163–72. doi: 10.1038/s41590-018-0276-y PMC6340744 30643263

[34] Mabbott NA Baillie JK Brown H Freeman TC Hume DA. An Expression Atlas of Human Primary Cells: Inference of Gene Function From Coexpression Networks. BMC Genomics (2013) 14:632. doi: 10.1186/1471-2164-14-632 24053356 PMC3849585

[35] Tibshirani R. The Lasso Method for Variable Selection in the Cox Model. Stat Med (1997) 16(4):385–95. doi: 10.1002/(SICI)1097-0258(19970228)16:4<385::AID-SIM380>3.0.CO;2-3 9044528

[36] Heagerty PJ Zheng Y. Survival Model Predictive Accuracy and ROC Curves. Biometrics (2005) 61(1):92–105. doi: 10.1111/j.0006-341X.2005.030814.x 15737082

[37] Kassambara A Kosinski M Biecek P Fabian S. Survminer: Drawing Survival Curves Using ‘Ggplot2’R Package Version 0.4.4 . Available at: https://CRAN.R-project.org/package=survminer.

[38] Yu G Wang LG Han Y He QY. Clusterprofiler: An R Package for Comparing Biological Themes Among Gene Clusters. OMICS (2012) 16(5):284–7. doi: 10.1089/omi.2011.0118 PMC3339379 22455463

[39] Gentleman RC Carey VJ Bates DM Bolstad B Dettling M Dudoit S. Bioconductor: Open Software Development for Computational Biology and Bioinformatics. Genome Biol (2004) 5(10):R80. doi: 10.1186/gb-2004-5-10-r80 15461798 PMC545600

[40] Newman AM Steen CB Liu CL Gentles AJ Chaudhuri AA Scherer F. Determining Cell Type Abundance and Expression From Bulk Tissues With Digital Cytometry. Nat Biotechnol (2019) 37(7):773–82. doi: 10.1038/s41587-019-0114-2 PMC6610714 31061481

[41] Rody A Holtrich U Pusztai L Liedtke C Gaetje R Ruckhaeberle E. T-Cell Metagene Predicts a Favorable Prognosis in Estrogen Receptor-Negative and HER2-Positive Breast Cancers. Breast Cancer Res (2009) 11(2):R15. doi: 10.1186/bcr2234 19272155 PMC2688939

[42] Hanzelmann S Castelo R Guinney J. GSVA: Gene Set Variation Analysis for Microarray and RNA-Seq Data. BMC Bioinf (2013) 14:7. doi: 10.1186/1471-2105-14-7 PMC3618321 23323831

[43] Gu Z Eils R Schlesner M. Complex Heatmaps Reveal Patterns and Correlations in Multidimensional Genomic Data. Bioinformatics (2016) 32(18):2847–9. doi: 10.1093/bioinformatics/btw313 27207943

[44] Yoshihara K Shahmoradgoli M Martinez E Vegesna R Kim H Torres-Garcia W. Inferring Tumour Purity and Stromal and Immune Cell Admixture From Expression Data. Nat Commun (2013) 4:2612. doi: 10.1038/ncomms3612 24113773 PMC3826632

[45] Mayakonda A Lin DC Assenov Y Plass C Koeffler HP. Maftools: Efficient and Comprehensive Analysis of Somatic Variants in Cancer. Genome Res (2018) 28(11):1747–56. doi: 10.1101/gr.239244.118 PMC6211645 30341162

[46] Chalmers ZR Connelly CF Fabrizio D Gay L Ali SM Ennis R. Analysis of 100,000 Human Cancer Genomes Reveals the Landscape of Tumor Mutational Burden. Genome Med (2017) 9(1):34. doi: 10.1186/s13073-017-0424-2 28420421 PMC5395719

[47] Thorsson V Gibbs DL Brown SD Wolf D Bortone DS Ou Yang TH. The Immune Landscape of Cancer. Immunity (2018) 48(4):812–30.e14. doi: 10.1016/j.immuni.2018.03.023 29628290 PMC5982584

[48] Schwarzer G. Meta: An R Package for Meta-Analysis. R News (2007) 7(3):40–5.

[49] Page DB Yuan J Redmond D Wen YH Durack JC Emerson R. Deep Sequencing of T-Cell Receptor DNA as a Biomarker of Clonally Expanded TILs in Breast Cancer After Immunotherapy. Cancer Immunol Res (2016) 4(10):835–44. doi: 10.1158/2326-6066.CIR-16-0013 PMC5064839 27587469

[50] Sims JS Grinshpun B Feng Y Ung TH Neira JA Samanamud JL. Diversity and Divergence of the Glioma-Infiltrating T-Cell Receptor Repertoire. Proc Natl Acad Sci USA (2016) 113(25):E3529–37. doi: 10.1073/pnas.1601012113 PMC4922177 27261081

[51] Han Y Li H Guan Y Huang J. Immune Repertoire: A Potential Biomarker and Therapeutic for Hepatocellular Carcinoma. Cancer Lett (2016) 379(2):206–12. doi: 10.1016/j.canlet.2015.06.022 26188280

[52] Luo Y Kong F Wang Z Chen D Liu Q Wang T. Loss of ASAP3 Destabilizes Cytoskeletal Protein ACTG1 to Suppress Cancer Cell Migration. Mol Med Rep (2014) 9(2):387–94. doi: 10.3892/mmr.2013.1831 24284654

[53] Chang YC Chiou J Yang YF Su CY Lin YF Yang CN. Therapeutic Targeting of Aldolase A Interactions Inhibits Lung Cancer Metastasis and Prolongs Survival. Cancer Res (2019) 79(18):4754–66. doi: 10.1158/0008-5472.CAN-18-4080 31358528

[54] Jung SH Lee HC Hwang HJ Park HA Moon YA Kim BC. Acyl-CoA Thioesterase 7 Is Involved in Cell Cycle Progression *via* Regulation of PKCzeta-P53-P21 Signaling Pathway. Cell Death Dis (2017) 8(5):e2793. doi: 10.1038/cddis.2017.202 28518146 PMC5584527

[55] Zhang L Xia H Xia K Liu X Zhang X Dai J. Selenium Regulation of the Immune Function of Dendritic Cells in Mice Through the ERK, Akt and RhoA/ROCK Pathways. Biol Trace Elem Res (2021) 199(9):3360–70. doi: 10.1007/s12011-020-02449-5 33107016

[56] Verma S Hoffmann FW Kumar M Huang Z Roe K Nguyen-Wu E. Selenoprotein K Knockout Mice Exhibit Deficient Calcium Flux in Immune Cells and Impaired Immune Responses. J Immunol (2011) 186(4):2127–37. doi: 10.4049/jimmunol.1002878 PMC3088479 21220695

[57] Jia Y Dai J Zeng Z. Potential Relationship Between the Selenoproteome and Cancer. Mol Clin Oncol (2020) 13(6):83. doi: 10.3892/mco.2020.2153 PMC7590431 33133596

[58] Wang Q Li XY Wan B Zhang J Sun R Zhou CY. Overexpression of Raf-1 Kinase Inhibitor Protein Inhibits Cell Invasion and Migration in Lung Cancer Cells Through Suppressing Epithelial-Mesenchymal Transition. Transl Cancer Res (2019) 8(6):2295–306. doi: 10.21037/tcr.2019.09.56 PMC8797464 35116982

[59] Ivagnes A Messaoudene M Stoll G Routy B Fluckiger A Yamazaki T. TNFR2/BIRC3-TRAF1 Signaling Pathway as a Novel NK Cell Immune Checkpoint in Cancer. Oncoimmunology (2018) 7(12):e1386826. doi: 10.1080/2162402X.2017.1386826 30524877 PMC6279330

[60] Tang D Liu H Zhao Y Qian D Luo S Patz EF Jr.. Genetic Variants of BIRC3 and NRG1 in the NLRP3 Inflammasome Pathway Are Associated With Non-Small Cell Lung Cancer Survival. Am J Cancer Res (2020) 10(8):2582–95. PMC7471354 32905523

[61] Ford HL Pardee AB. Cancer and the Cell Cycle. J Cell Biochem (1999) Suppl 32-33:166–72. doi: 10.1002/(SICI)1097-4644(1999)75:32+<166::AID-JCB20>3.0.CO;2-J 10629116

[62] Barnes TA Amir E. HYPE or HOPE: The Prognostic Value of Infiltrating Immune Cells in Cancer. Br J Cancer (2017) 117(4):451–60. doi: 10.1038/bjc.2017.220 PMC5558691 28704840

[63] Bonaventura P Shekarian T Alcazer V Valladeau-Guilemond J Valsesia-Wittmann S Amigorena S. Cold Tumors: A Therapeutic Challenge for Immunotherapy. Front Immunol (2019) 10:168. doi: 10.3389/fimmu.2019.00168 30800125 PMC6376112

[64] Roseweir AK Powell A Horstman SL Inthagard J Park JH McMillan DC. Src Family Kinases, HCK and FGR, Associate With Local Inflammation and Tumour Progression in Colorectal Cancer. Cell Signal (2019) 56:15–22. doi: 10.1016/j.cellsig.2019.01.007 30684564

[65] Salmond RJ Filby A Qureshi I Caserta S Zamoyska R. T-Cell Receptor Proximal Signaling *via* the Src-Family Kinases, Lck and Fyn, Influences T-Cell Activation, Differentiation, and Tolerance. Immunol Rev (2009) 228(1):9–22. doi: 10.1111/j.1600-065X.2008.00745.x 19290918

[66] Garrido F Aptsiauri N. Cancer Immune Escape: MHC Expression in Primary Tumours Versus Metastases. Immunology (2019) 158(4):255–66. doi: 10.1111/imm.13114 PMC6856929 31509607

