# Supplementary material for: Identification and Validation of a Novel Signature Based on NK Cell Marker Genes to Predict Prognosis and Immunotherapy Response in Lung Adenocarcinoma by Integrated Analysis of Single-Cell and Bulk RNA-Sequencing
Source: Front Immunol. 2022 Jun 10;13:850745. doi: 10.3389/fimmu.2022.850745 (PMC9231585; doi:10.3389/fimmu.2022.850745)
Supplement: Supplementary file 1 [file DataSheet_1.pdf]

Supplementary Table 1. Eight datasets used for analysis in our study

ID	Platform	Type	Samples (used in this study)	Samples (Total)	URLs
TCGA-LUAD	Illumina HiSeq 2000	RNA-seq	500	585	https://xenabrowser.net/
GSE131907	GPL16791(Illumina HiSeq 2500)	RNA-seq	11	58	https://www.ncbi.nlm.nih.gov/geo/query/acc.cgi?acc=GSE131907
GSE30219	GPL570 (Affymetrix HG U133 Plus 2.0)	Microarray	83	307	https://www.ncbi.nlm.nih.gov/geo/query/acc.cgi?acc=GSE30219
GSE3141	GPL570 (Affymetrix HG U133 Plus 2.0)	Microarray	58	111	https://www.ncbi.nlm.nih.gov/geo/query/acc.cgi?acc=GSE3141
GSE50081	GPL570 (Affymetrix HG U133 Plus 2.0)	Microarray	127	181	https://www.ncbi.nlm.nih.gov/geo/query/acc.cgi?acc=GSE50081
GSE26939	GPL9053 (Agilent-4X44K)	Microarray	115	116	https://www.ncbi.nlm.nih.gov/geo/query/acc.cgi?acc=GSE26939
GSE72094	GPL15048 (Affymetrix Rosetta/Merck Human RSTA 2.0)	Microarray	398	442	https://www.ncbi.nlm.nih.gov/geo/query/acc.cgi?acc=GSE72094
GSE31210	GPL570 (Affymetrix HG U133 Plus 2.0)	Microarray	226	246	https://www.ncbi.nlm.nih.gov/geo/query/acc.cgi?acc=GSE31210

Supplementary Table 2. NK cell marker genes identified from GSE131907

No.	Gene	No.	Gene	No.	Gene	No.	Gene
1	TPSB2	32	SAMSN1	63	STX3	94	CYTIP
2	TPSAB1	33	ADRB2	64	HLA-A	95	ID2
3	CPA3	34	FOSB	65	RENBP	96	EVL
4	HPGDS	35	PTGS1	66	CD69	97	RGS10
5	MS4A2	36	TMEM233	67	LEO1	98	PTGS2
6	CTSG	37	FCER1G	68	ACOT7	99	NFKBIZ
7	GATA2	38	LAT2	69	CLIC1	100	ITM2C
8	HPGD	39	CNRIP1	70	SELK	101	HLA-DPA1
9	RGS13	40	RAB27B	71	LSP1	102	CPM
10	C1orf186	41	BTK	72	STMN1	103	RGS2
11	VWA5A	42	SRGN	73	HLA-C	104	CTNNBL1
12	LTC4S	43	CALB2	74	MSRA	105	LTB
13	SLC18A2	44	BACE2	75	GPR65	106	PEBP1
14	CLU	45	H3F3B	76	ANKRD28	107	CD2
15	NFKBIA	46	FTH1	77	BTG1	108	CD82
16	LAPTM4A	47	SMYD3	78	SDCBP	109	BEX4
17	CD9	48	SVOPL	79	TMEM176B	110	ISG20
18	KIT	49	VIM	80	ARHGEF6	111	CD3E
19	FCER1A	50	MITF	81	TRAC	112	ALOX5
20	DUSP6	51	SLC45A3	82	TSC22D1	113	TRBC2
21	LMNA	52	STXBP6	83	CORO1A	114	FYB
22	MAOB	53	CSF1	84	PLIN2	115	ANXA2
23	IL1RL1	54	GRAP2	85	HLA-DRB1	116	BIRC3
24	ANXA1	55	HLA-B	86	TNFSF10	117	NSMCE1
25	LMO4	56	HINT1	87	HLA-DPB1	118	ARHGAP18
26	HDC	57	ACTG1	88	ASAH1	119	HLA-DQB1
27	GLUL	58	ZFP36L2	89	PRDX6	120	PPP1R15A
28	CD63	59	CD74	90	GALC	121	HLA-DRA
29	CAPG	60	IL32	91	CD3D	122	CCL5
30	GCSAML	61	CXCR4	92	BHLHE40	123	DDIT4
31	ACSL4	62	HES1	93	RGCC	124	ITGB2

No.	Gene	No.	Gene	No.	Gene
125	RAB32	156	CD3G	187	APOC1
126	ELL2	157	DNAJB1	188	CTSB
127	HLA-DQA1	158	ARID5B	189	APOE
128	DDAH2	159	RORA		
129	VAPA	160	LIMD2		
130	GYPC	161	TWISTNB		
131	LCK	162	CLEC2D		
132	YPEL5	163	TUBA1A		
133	RGS1	164	HLA-DMB		
134	HSD17B12	165	NKG7		
135	TRBC1	166	SGK1		
136	GLIPR1	167	HOPX		
137	IL7R	168	DUSP4		
138	GPR183	169	COTL1		
139	HLA-DMA	170	SOD2		
140	KDM6B	171	FABP5		
141	AHR	172	CTSS		
142	EZR	173	IGKC		
143	ACAP1	174	CTSZ		
144	EML4	175	IGLC2		
145	CTSH	176	CCL4		
146	GZMA	177	SERPINA1		
147	TUBA4A	178	S100A9		
148	CD7	179	IGHA1		
149	PTPRC	180	IGHG3		
150	STK17A	181	C1QB		
151	S100A10	182	IGLC3		
152	IL2RG	183	IGHG1		
153	ARL4C	184	C1QA		
154	SPOCK2	185	IGHG4		
155	GADD45B	186	LYZ		

Supplementary Table 3. Overall survival associated gene list in LUAD patients from TCGA

No.	Gene	HR	*p* value	No.	Gene	HR	*p* value
1	HPGDS	0.667	0.001	14	SELENOK	0.129	0.006
2	MS4A2	0.702	0.004	15	PEBP1	0.097	0.004
3	CTSG	0.733	0.005	16	BEX4	0.358	0.000
4	RGS13	0.498	0.001	17	ANXA2	6.089	0.003
5	SLC18A2	0.638	0.002	18	BIRC3	1.952	0.005
6	KIT	0.700	0.008	19	DDIT4	2.971	0.002
7	HDC	0.674	0.004	20	TRBC1	0.629	0.004
8	GCSAML	0.592	0.006	21	HLA-DMA	0.359	0.001
9	ADRB2	0.673	0.005	22	ACAP1	0.525	0.002
10	GRAP2	0.601	0.004	23	CTSH	0.301	0.000
11	ACTG1	34.327	0.002	24	S100A10	5.692	0.003
12	ACOT7	3.156	0.004	25	HOPX	0.649	0.006
13	CLIC1	20.077	0.002				

Supplementary Table 4. 124 genes that closely correlated with the signature

No.	Gene	R	*p* value	No.	Gene	R	*p* value
1	SLC2A1	0.519	6.88E-36	39	PRC1	0.429	8.90E-24
2	ANLN	0.509	2.74E-34	40	FOXM1	0.427	1.26E-23
3	KCMF1	0.491	1.05E-31	41	CMAS	0.426	1.87E-23
4	SH2D5	0.489	1.89E-31	42	B3GNT5	0.426	2.06E-23
5	ERO1A	0.485	8.19E-31	43	MAPK6	0.426	2.07E-23
6	ARNTL2	0.484	9.21E-31	44	VANGL1	0.425	2.15E-23
7	HMGA1	0.481	2.29E-30	45	LYAR	0.424	2.91E-23
8	GAPDH	0.477	8.54E-30	46	CRYBG2	0.424	2.97E-23
9	BCAR3	0.474	2.51E-29	47	ACOT7	0.424	3.30E-23
10	FOSL1	0.467	1.95E-28	48	PRR11	0.424	3.39E-23
11	CREG2	0.461	9.99E-28	49	PSMD2	0.424	2.86E-23
12	LINC02178	0.461	1.18E-27	50	KNL1	0.422	5.43E-23
13	PLIN3	0.455	7.27E-27	51	RAD51	0.422	5.17E-23
14	MYO1E	0.454	7.55E-27	52	INTS13	0.421	7.53E-23
15	DSG2	0.454	8.01E-27	53	LYPD3	0.421	6.44E-23
16	EXO1	0.452	1.51E-26	54	KRT18	0.421	5.97E-23
17	LDHA	0.451	1.92E-26	55	ORC1	0.421	6.99E-23
18	PKM	0.451	2.32E-26	56	FBXO45	0.42	8.74E-23
19	GJB3	0.449	3.65E-26	57	KIF14	0.42	7.87E-23
20	PITX3	0.447	6.24E-26	58	GALNT2	0.418	1.43E-22
21	RHOF	0.446	9.44E-26	59	CCNA2	0.418	1.41E-22
22	AP1S3	0.446	7.90E-26	60	KPNA2	0.418	1.29E-22
23	PLK1	0.445	9.89E-26	61	AP000695.2	0.418	1.51E-22
24	LINC00941	0.445	1.03E-25	62	ITGB1-DT	0.418	1.28E-22
25	LAMC2	0.442	2.82E-25	63	NCAPG	0.416	2.22E-22
26	CD109	0.442	2.21E-25	64	CEP55	0.416	2.72E-22
27	ECT2	0.441	3.60E-25	65	NTSR1	0.415	2.86E-22
28	ARHGAP11A	0.44	4.54E-25	66	TPX2	0.415	3.19E-22
29	AHNAK2	0.433	3.07E-24	67	AC027237.1	0.415	3.45E-22
30	RGS20	0.432	4.06E-24	68	CTSV	0.414	4.15E-22
31	EHBP1	0.431	5.50E-24	69	VASP	0.413	5.14E-22
32	TK1	0.43	5.74E-24	70	DEPDC1	0.413	4.79E-22
33	PSMD12	0.43	5.91E-24	71	AC099850.3	0.413	5.66E-22
34	VDAC1	0.43	6.45E-24	72	TEAD4	0.412	6.28E-22
35	PVR	0.429	7.96E-24	73	KIF4A	0.412	5.96E-22
36	RRM2	0.429	8.79E-24	74	MKI67	0.412	6.90E-22
37	SHCBP1	0.429	9.35E-24	75	CFL1	0.412	6.52E-22
38	BZW1	0.429	9.07E-24	76	DTL	0.411	9.17E-22

No.	Gene	R	*p* value	No.	Gene	R	*p* value
77	DIAPH3	0.41	9.67E-22	101	SELENBP1	-0.402	8.05E-21
78	SMS	0.41	1.09E-21	102	N4BP2L1	-0.403	6.45E-21
79	SLC16A3	0.41	1.04E-21	103	GNG7	-0.403	6.41E-21
80	TUBA1C	0.408	1.86E-21	104	LINC00892	-0.404	4.18E-21
81	KRT18P28	0.408	1.57E-21	105	MTURN	-0.405	3.63E-21
82	ITGA5	0.408	1.89E-21	106	AC087752.3	-0.406	2.74E-21
83	BUB1B	0.408	1.60E-21	107	SLC25A42	-0.407	2.21E-21
84	CKAP2L	0.407	2.36E-21	108	DAPK2	-0.407	2.26E-21
85	ERCC6L	0.407	2.51E-21	109	METTL7A	-0.409	1.26E-21
86	SKA3	0.406	3.16E-21	110	PPM1M	-0.41	1.10E-21
87	ADIPOR2	0.406	2.71E-21	111	BTG2	-0.41	1.17E-21
88	PSMD1	0.405	3.59E-21	112	FAM117A	-0.411	8.84E-22
89	SMCO2	0.405	3.73E-21	113	PPIAP39	-0.411	9.16E-22
90	EIF4A3	0.404	4.42E-21	114	CIRBP	-0.413	5.36E-22
91	KIF18A	0.404	4.81E-21	115	TMEM125	-0.421	6.27E-23
92	AL161668.1	0.404	4.94E-21	116	C16orf89	-0.422	4.94E-23
93	ITGB1	0.403	6.29E-21	117	FRZB	-0.425	2.42E-23
94	CDC6	0.402	7.67E-21	118	PTGDS	-0.426	1.94E-23
95	KIF2C	0.402	8.26E-21	119	CMAHP	-0.437	9.35E-25
96	LRRC59	0.402	7.14E-21	120	ZNF540	-0.438	7.91E-25
97	KRT8	0.402	7.26E-21	121	SFTA3	-0.439	5.22E-25
98	AUNIP	0.402	6.88E-21	122	CXCL17	-0.45	2.35E-26
99	ERLIN1	0.401	8.93E-21	123	ACSS1	-0.459	2.31E-27
100	AC034223.2	0.401	9.23E-21	124	NKX2-1	-0.46	1.73E-27

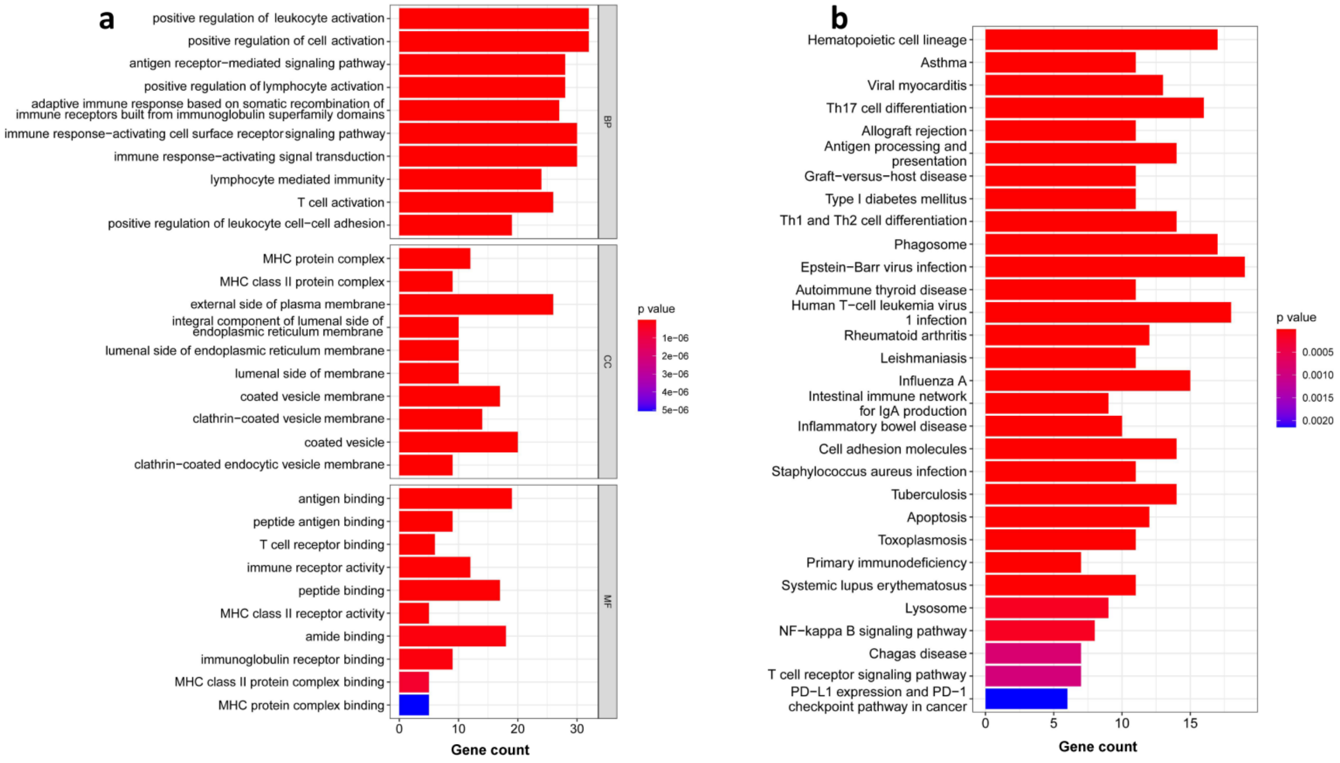

Supplementary Figure 1. Functional enrichment analysis of NK cell marker genes of LUAD. GO (a) and KEGG (b) analysis of the NK cell marker genes.

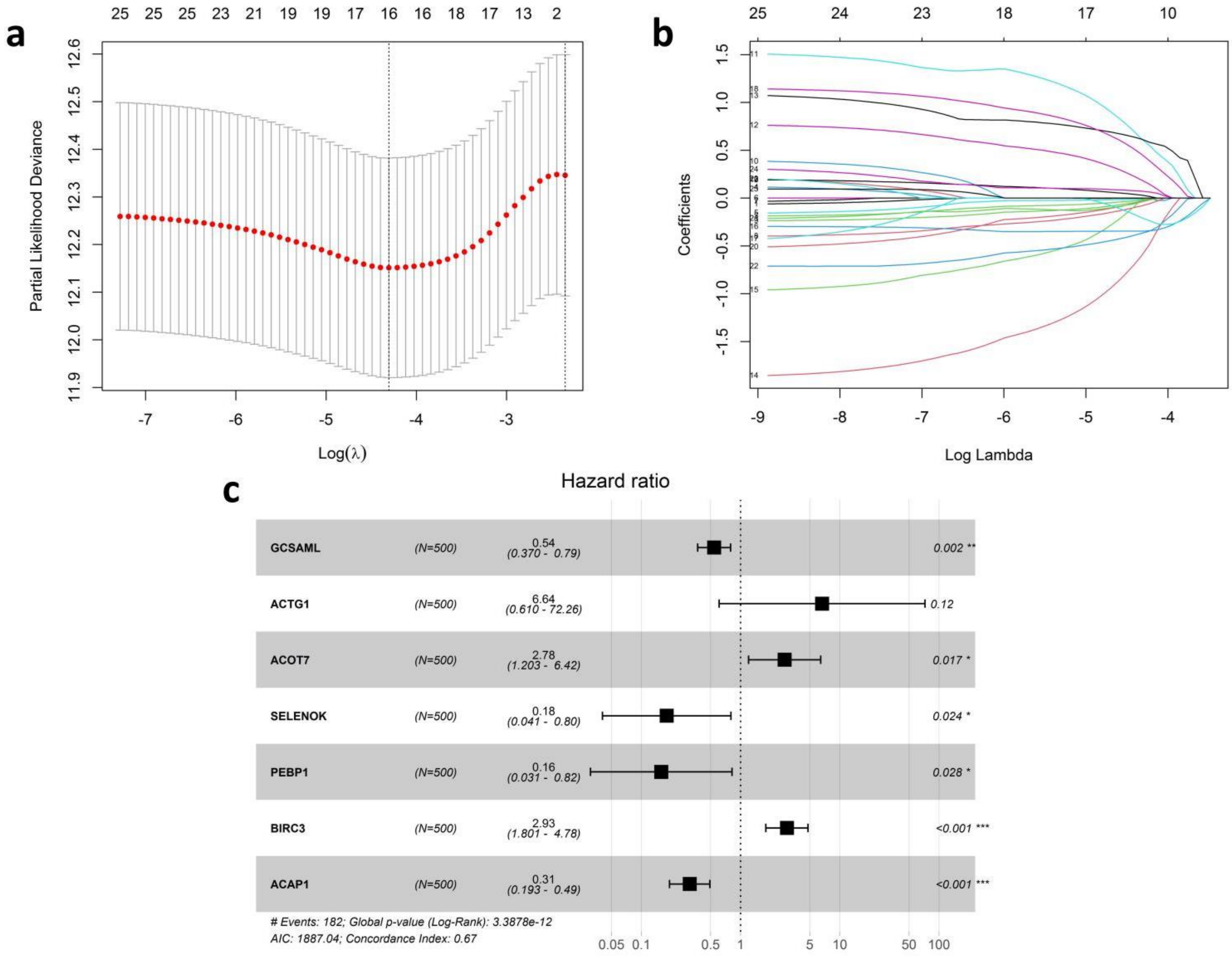

Supplementary Figure 2. Construction of NK cell marker genes signature in TCGA LUAD cohorts. (a) LASSO coefficient profiles of the 25 prognostic genes. (b) 10-fold cross-validation for tuning parameter selection in the LASSO model. (c) Multivariable Cox proportional hazards regression analysis of the 7 prognostic genes.

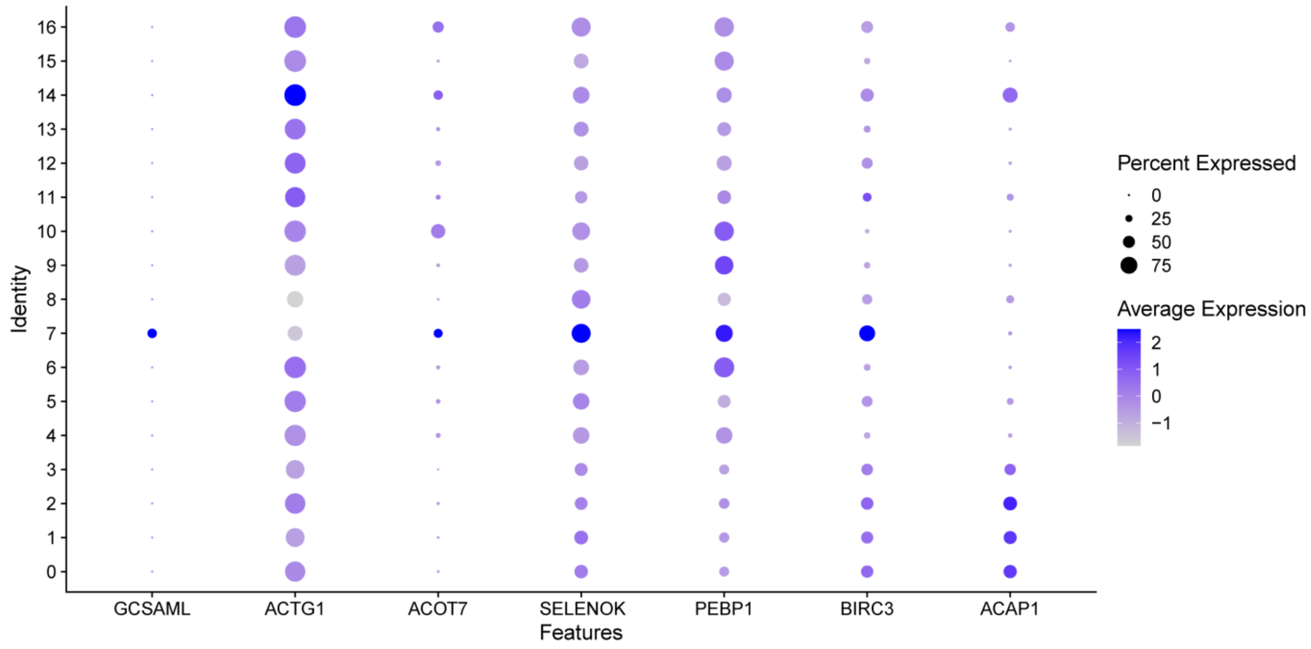

Supplementary Figure 3. Bubble plot showing the expression of NK cell marker genes included in the risk score model in various cell types. Bubble intensity of colour indicates the average expression in a particular cluster and bubble size represents the percent of cells expressing the gene in that cluster. Cluster 7 represents NK cells.

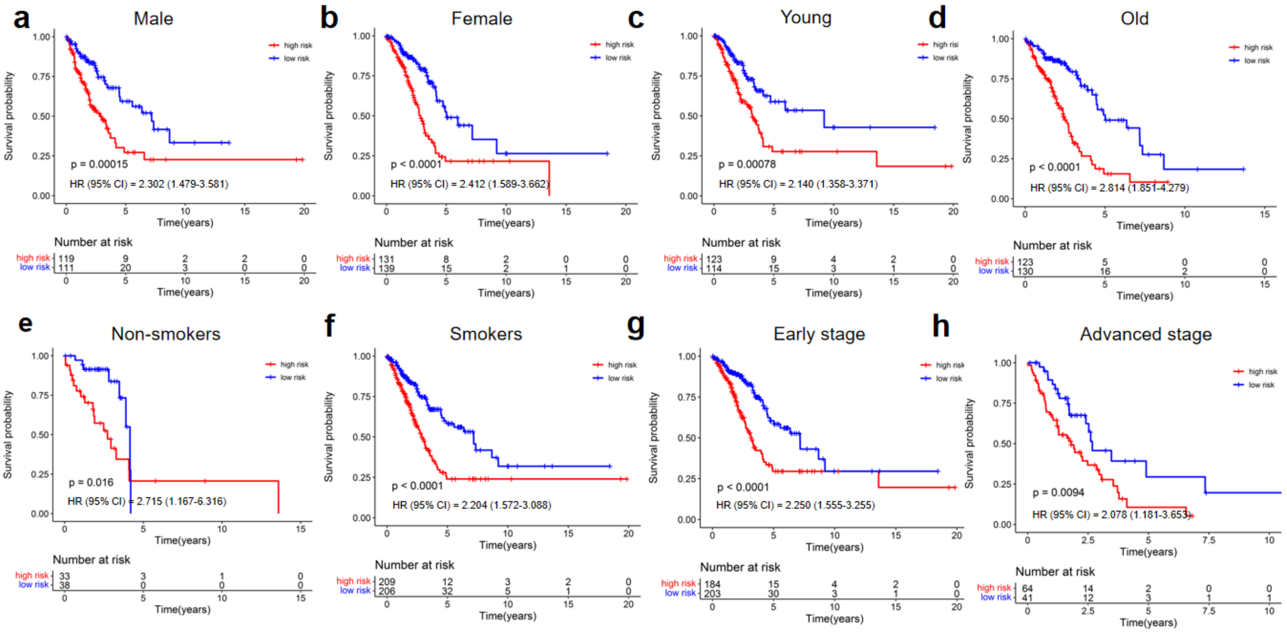

Supplementary Figure 4. Validation of the prognostic predictive ability of NK cell marker genes signature in different clinical subgroups. Kaplan–Meier curves of overall survival in male (a), female (b), young (c), old (d), non-smokers (e), smokers (f), early stage (g) and advanced stage (h) patients based on risk score in TCGA LUAD cohort.

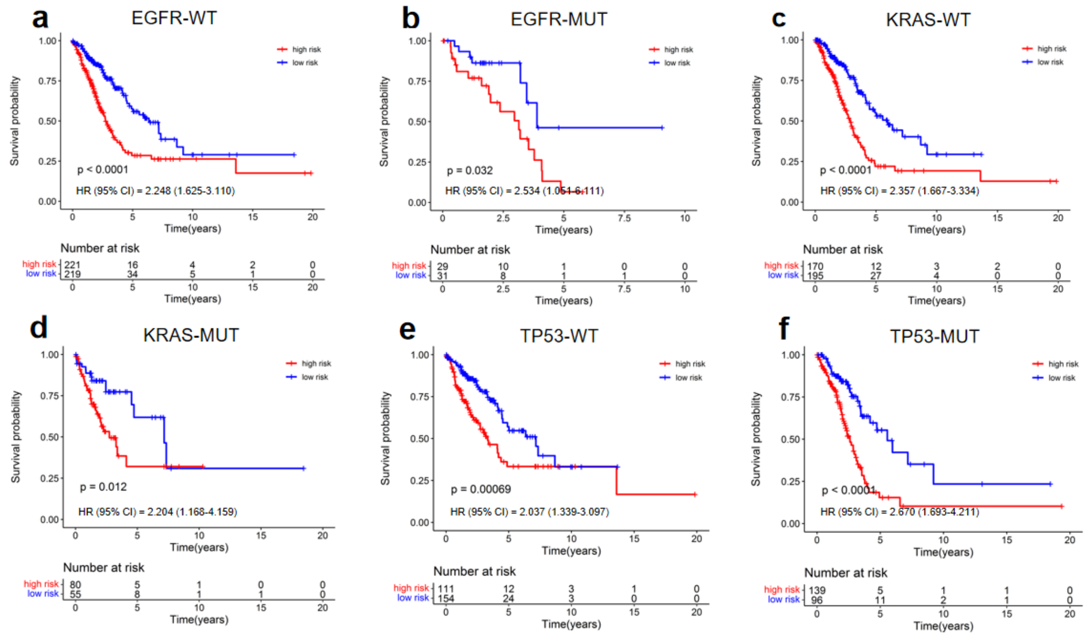

Supplementary Figure 5. Validation of the prognostic predictive ability of NK cell marker genes signature with different mutation status. Kaplan–Meier survival analysis of overall survival for patients with EGFR-WT (A), EGFR-MUT (B), KRAS-WT (C), KRAS-MUT (D), TP53-WT (E) and TP53-MUT (F) based on risk score in TCGA cohort. WT, wild-type; MUT, mutation.

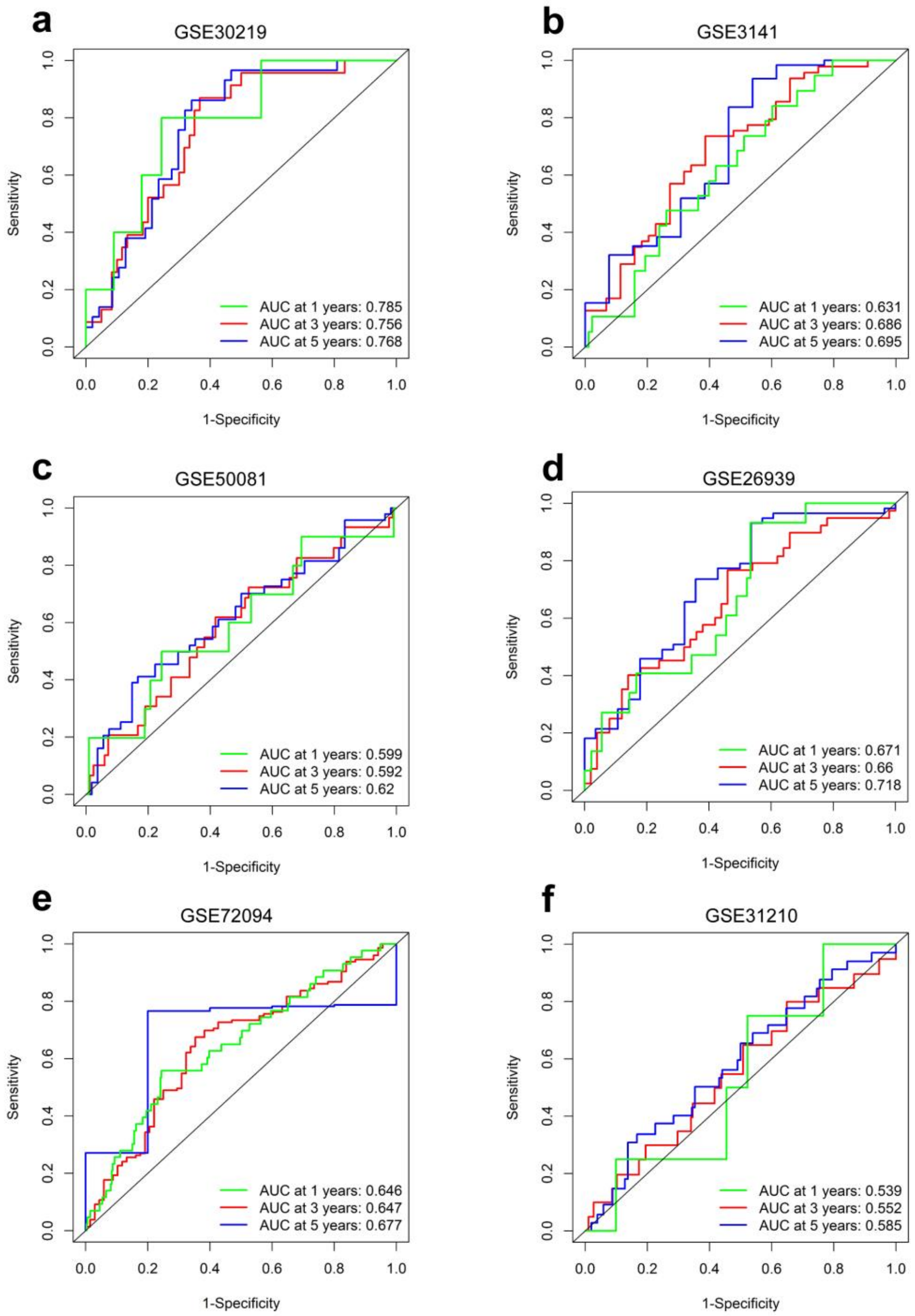

Supplementary Figure 6. Time-dependent ROC curves of risk score for predicting overall survival in GSE30219 cohort (a), GSE3141 cohort (b), GSE50081 cohort (c), GSE26939 cohort (d), GSE72094 cohort (e) and GSE31210 cohort (f).

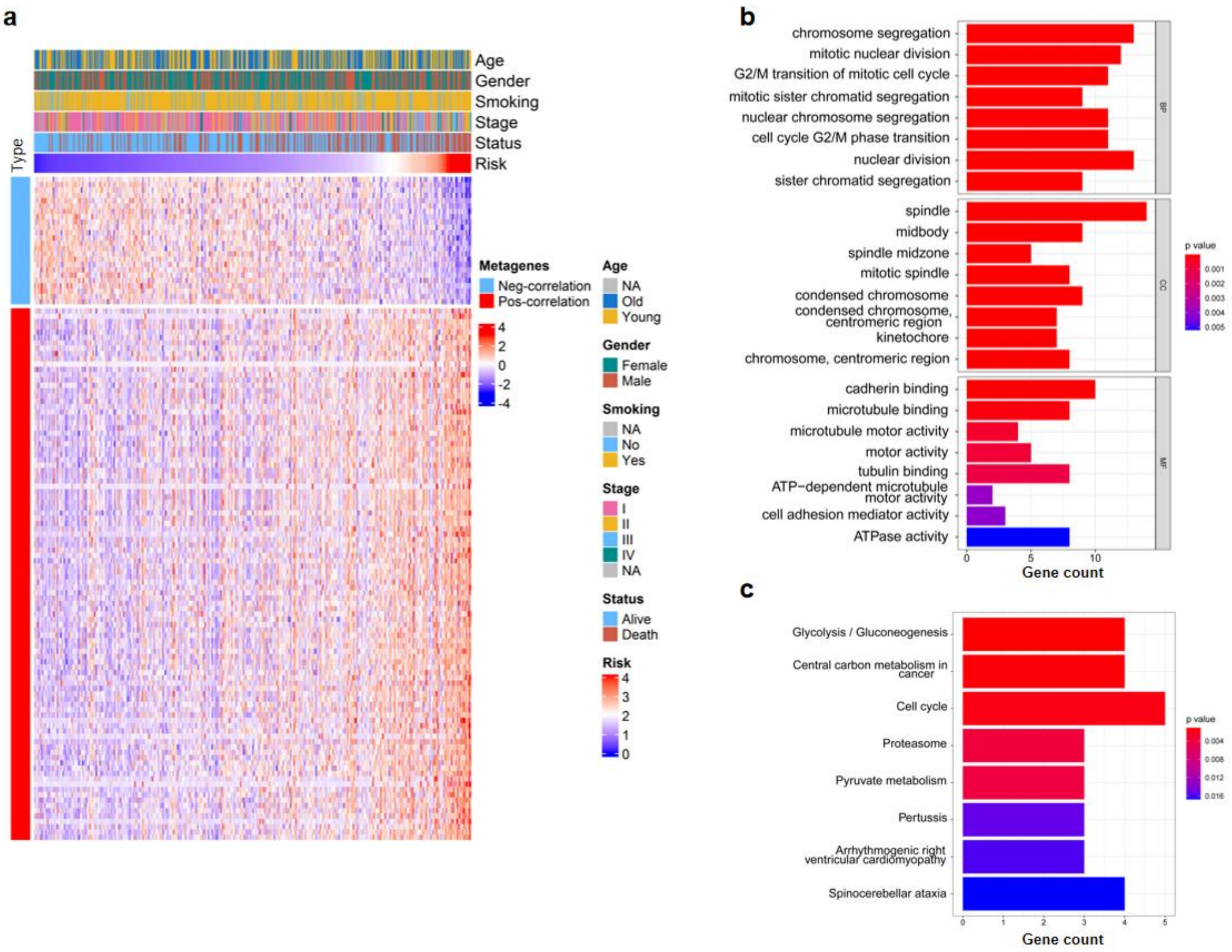

Supplementary Figure 7. Functional enrichment analysis of the NKCMGS related genes. (a) Heatmap showed the expression characteristics of 124 genes that closely correlated with the signature (Pearson |R| > 0.4, P < 0.05). (b) Barplot of GO analysis of the 124 genes. (c) Barplot of KEGG analysis of the 124 genes.

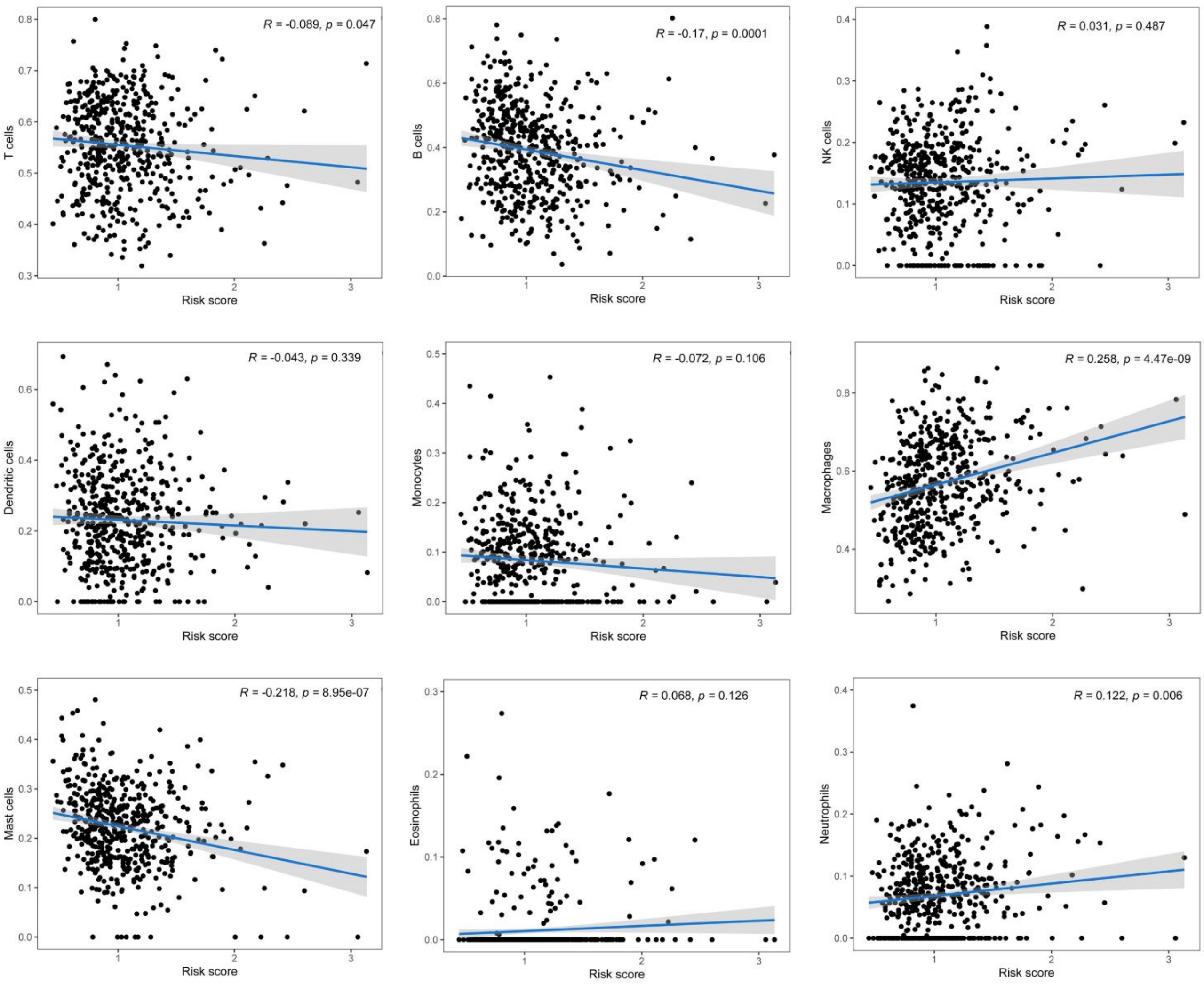

Supplementary Figure 8. The correlation analysis between estimated immune cell infltration level and risk score.

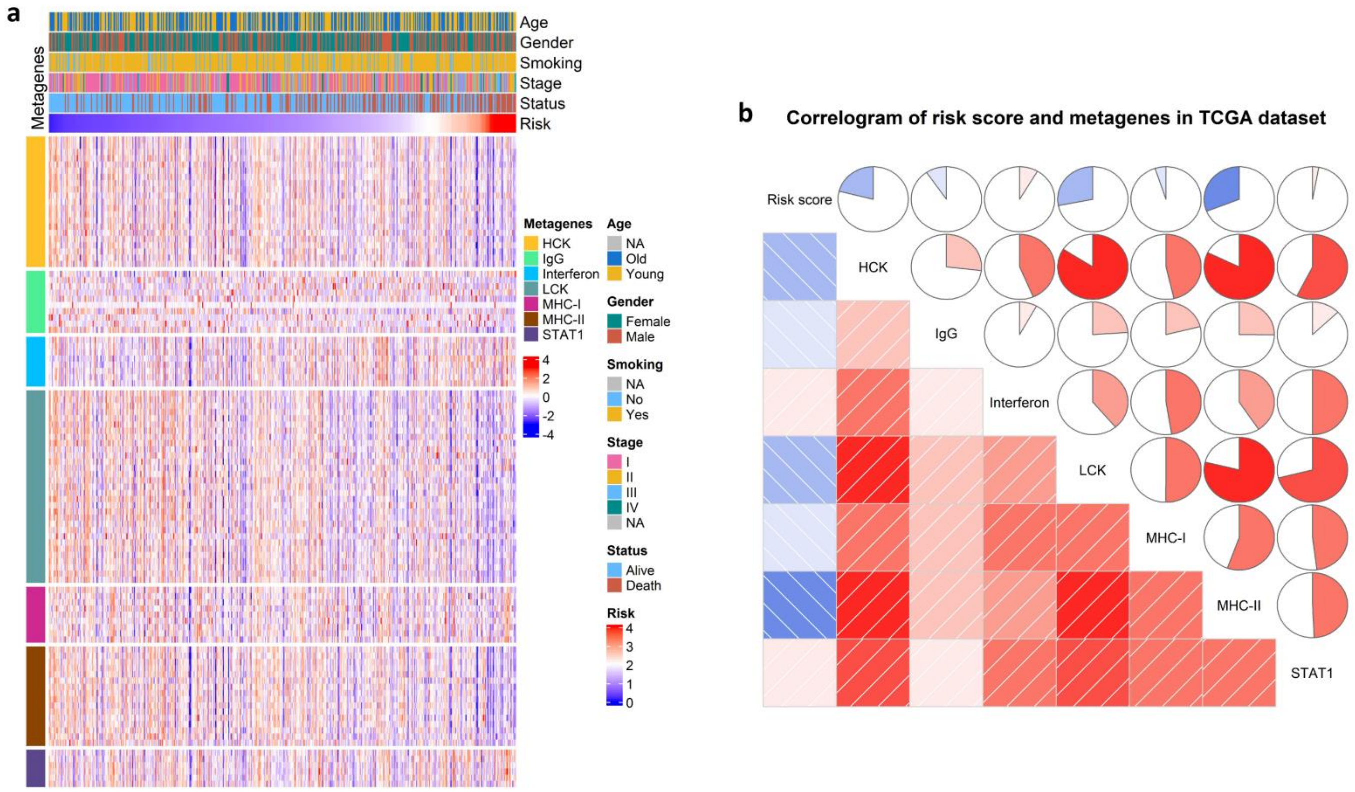

Supplementary Figure 9. Relationship between the NKCMGS and inflammatory metagenes in TCGA LUAD cohort. (a) Heatmap showed the expression characteristics of 7 clusters of metagenes. (b) The plot of Correlogram was produced by using Pearson R-value between metagenes and risk scores.
